# The effect of health financing systems on health system outcomes: A cross‐country panel analysis

**DOI:** 10.1002/hec.4635

**Published:** 2022-12-08

**Authors:** Jacopo Gabani, Sumit Mazumdar, Marc Suhrcke

**Affiliations:** ^1^ Centre for Health Economics University of York York UK; ^2^ Department of Economics and Related Studies University of York York UK; ^3^ Luxembourg Institute of Socio‐Economic Research (LISER) Esch‐sur‐Alzette Luxembourg

**Keywords:** health expenditure, health financing, health system, social health insurance, universal health coverage

## Abstract

Several low‐ and middle‐income countries are considering health financing system reforms to accelerate progress toward universal health coverage (UHC). However, empirical evidence of the effect of health financing systems on health system outcomes is scarce, partly because it is difficult to quantitatively capture the ‘health financing system’. We assign country‐year observations to one of three health financing systems (i.e., predominantly out‐of‐pocket, social health insurance (SHI) or government‐financed), using clustering based on out‐of‐pocket, contributory SHI and non‐contributory government expenditure, as a percentage of total health expenditures. We then estimate the effect of these different systems on health system outcomes, using fixed effects regressions. We find that transitions from OOP‐dominant to government‐financed systems improved most outcomes more than did transitions to SHI systems. Transitions to government financing increases life expectancy (+1.3 years, *p* < 0.05) and reduces under‐5 mortality (−8.7%, *p* < 0.05) and catastrophic health expenditure incidence (−3.3 percentage points, *p* < 0.05). Results are robust to several sensitivity tests. It is more likely that increases in non‐contributory government financing rather than SHI financing improve health system outcomes. Notable reasons include SHI's higher implementation costs and more limited coverage. These results may raise a warning for policymakers considering SHI reforms to reach UHC.

## 1 INTRODUCTION

Universal health coverage (UHC) captures the ambition that the entire population in a given jurisdiction receive the quality health services they need, without suffering financial hardship, regardless of socio‐economic conditions (World Health Organization, 2020). Several countries are currently considering health financing system (HFS) reforms to accelerate progress toward UHC (Savedoff & Yazbeck, 2020; Yazbeck et al., 2020). These reforms may entail the expansion of non‐contributory government financing arrangements (e.g., Brazil, Bolivia), or the introduction and/or expansion of contributory social health insurance (SHI) arrangements (e.g., Ghana, Ethiopia). In this paper, SHI financing refers to health expenditures channelled via SHI agencies, implying that a contribution is required to access services, irrespective of whether the contribution is subsidized by the government or not. Government financing refers to any other non‐contributory public health expenditure, that is, where access to services is automatic, not linked to contributions, and usually based on citizenship or residency status. In either case, the aim is to increase pooled public health expenditure and to transition away from out‐of‐pocket (OOP) private health expenditure (World Health Organization, 2019) toward UHC. HFS reforms entail substantial long‐term administrative efforts (e.g., setting up new laws and functional agencies), and may impact financial risk protection and population health for years to come.

Despite the importance of HFS as a major factor for achieving UHC, there is scarce empirical cross‐country evidence on the impact of HFSs on health system outcomes. Two important but regionally focused studies (on OECD and Eastern European countries) from more than a decade ago concluded that introducing SHI led to no improvement or even to a deterioration of health outcomes, while having increased costs (Wagstaff, 2009a; Wagstaff and Moreno‐Serra, 2009a). A common issue in these studies is that a country's HFS, depending on existing laws, could only be classified as either “tax‐based” or “SHI”. By allowing only these two classifications, countries financed predominantly by OOP expenditures were (mis‐)classified as either tax‐based or SHI. In addition, only the effects of transitioning from tax‐based to SHI HFS were examined, thus ignoring the potential effects of transitioning from predominantly OOP to either tax‐based or SHI HFSs. Another global study found that (proportional) increases in expenditure in contributory SHI and non‐contributory government financing are positively correlated with service coverage indicators, but only non‐contributory government financing is correlated with improvements in financial risk protection (Wagstaff & Neelsen, 2020). This study investigated the association of HFS with financial risk protection and service coverage but not health status, controlled only for GDP per capita, and most importantly did not investigate transitions from OOP to either SHI or government financing predominant HFS, which is arguably the decision commonly faced by policymakers when contemplating potential paths toward UHC. Other, broadly related studies have investigated the impact of public health expenditure on health system outcomes, mostly finding a positive effect (Nakamura et al., 2016), yet without differentiating public health expenditures into government or SHI financing sources. Finally, a recent systematic review of relevant country case‐studies concludes that public health insurance, defined as SHI and community‐based health insurance, appears to reduce financial risk protection (Erlangga et al., 2019). However, it is not clear whether this effect is applicable to the entire populations of SHI‐countries, or to SHI beneficiaries alone.

In this paper, we seek to assess the impact of different HFSs on health system outcomes (i.e., health status, financial risk protection and utilization (Kutzin, 2008)), and on health expenditures, with a view to informing decisions about potential transitions to either contributory SHI or non‐contributory government financing, aimed at accelerating progress toward UHC. We also shed light on potential contextual factors likely to affect the impact of HFSs.

We find that transitions from OOP‐ to SHI‐predominant HFS resulted in increased total health expenditure. However, transitions to government‐predominant HFS resulted in greater immunization coverage, and improved health system outcomes (life expectancy, under‐5 mortality and incidence of catastrophic health expenditure). As potential reasons, we discuss the role of (higher) costs for implementing SHI, its benefits being contribution‐linked, the tendency to favor secondary/tertiary care expenditures, and SHI's limited ability to decrease OOP expenditures. We also detect a role for contextual factors: in particular, increases in informal sector size diminish the effects of HFS on most health system outcomes. Other contextual factors considered (GDP per capita, governance) also act as effect modifiers of HFS, albeit to a lesser extent.

Endogeneity, driven by reverse causality (e.g., as countries with low financial risk protection may be more likely to introduce SHI) and omitted variable bias, is a central challenge in all studies investigating the association between HFS, health expenditure and health system outcomes (Nakamura et al., 2016), and our study is no exception in this regard. We seek to address endogeneity via fixed effects regressions, exploiting the variation in HFS generated by the health financing transition, which allows controlling for the influence of unobservable or unmeasured time‐invariant factors. As our results are robust to most, but not all, different specifications and outcomes, concerns regarding endogeneity driven by reverse causality are not completely resolved. For this reason, we do not claim to provide entirely causal evidence.

This paper contributes to the literature in several ways. First, we refine the classification of HFS by using a machine‐learning, data‐driven approach, which allows us to distinguish contributory SHI‐, non‐contributory government‐, and OOP‐predominant HFSs. Second, we examine the separate effects of transitions *from* OOP‐predominant *to* SHI‐ *or* government‐predominant HFSs. This is an advance on previous studies that commonly considered public health expenditure as a bundled aggregate, irrespectively of its specific financing nature (Filmer & Pritchett, 1999; Moreno‐Serra & Smith, 2015; Nakamura et al., 2016; Rajkumar & Swaroop, 2008), and on studies that did not model transitions from OOP‐ to SHI‐ or government‐predominant HFSs (Wagstaff, 2009a; Wagstaff & Moreno‐Serra, 2009a; Wagstaff & Neelsen, 2020). We also use panel data across more country‐years than previous HFS studies, and—in order to reduce omitted variable bias—we take into account the potential role of multiple contextual factors that were used in the public health expenditure literature (Nakamura et al., 2016), but were neglected in previous SHI‐related studies (Wagstaff, 2009a; Wagstaff & Moreno‐Serra, 2009a; Wagstaff & Neelsen, 2020). Finally, we provide more depth to the conclusion that “context matters”, by empirically investigating interactions between contextual factors (e.g., informal sector size) and HFS transitions.

While these results should not be taken to imply that non‐contributory government‐predominant HFSs are *always* ‘better’ than contributory SHI‐predominant systems, they may raise a warning to policymakers favoring the path of SHI to accelerate progress toward UHC, while reassuring those aiming for expansions of non‐contributory government‐financed systems.

## 2 HEALTH FINANCING SYSTEMS (HFS) AND HYPOTHETICAL EFFECTS ON HEALTH SYSTEM OUTCOMES

**FIGURE 1:**
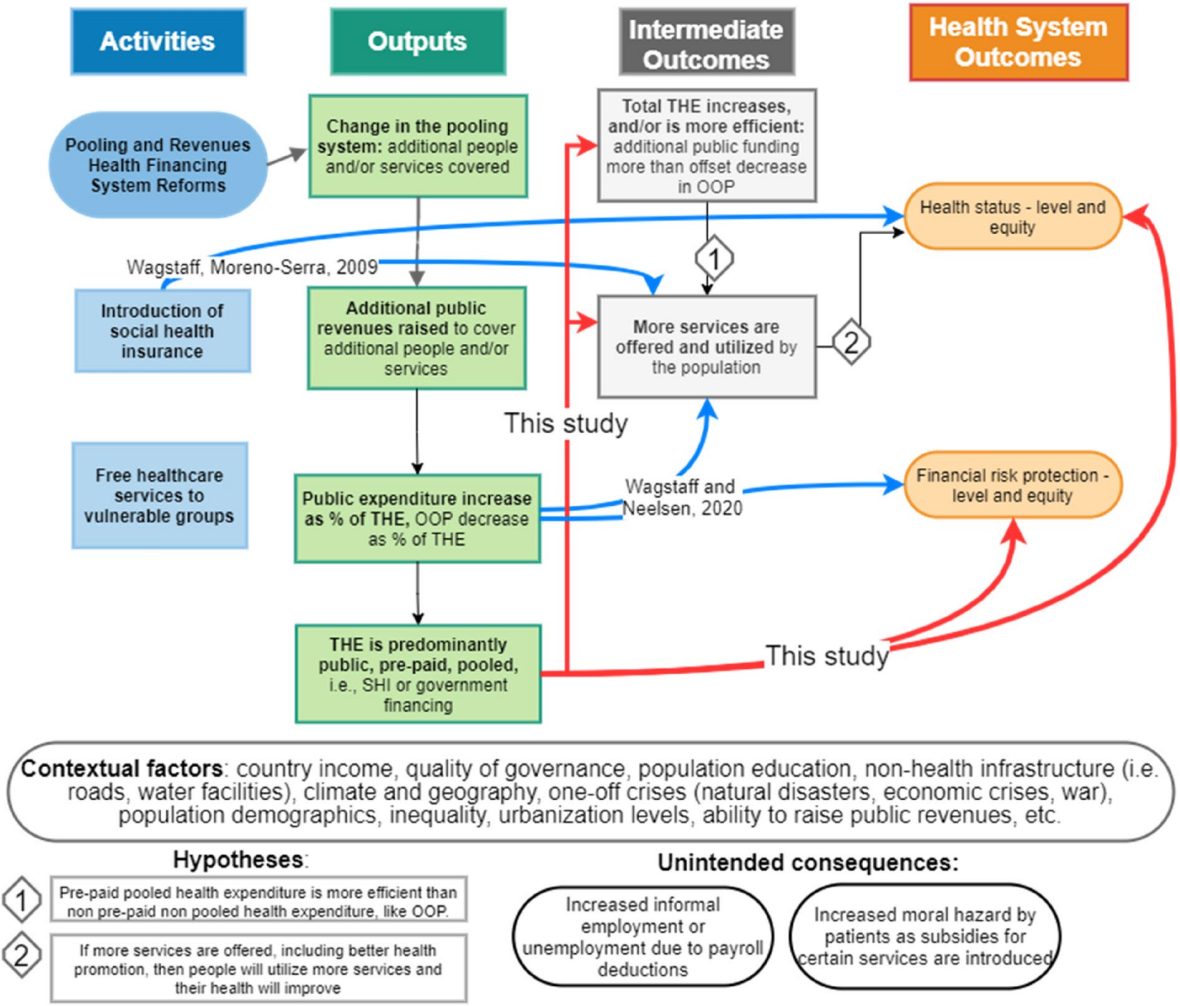
Conceptual framework. Source: authors' elaboration, expanding frameworks presented in (Kutzin, 2008). The conceptual framework follows a logic model representation. Black lines represent potential causal pathways between HFS and health system outcomes; numbers attached to black lines refer to hypotheses listed in the “Hypotheses” section. The two hypotheses and two unintended consequences noted in the figure are not exhaustive. Red lines represent the pathways investigated by this study. Blue lines represent pathways investigated by existing cross‐country regression studies: Wagstaff and Moreno‐Serra, 2009, and Wagstaff and Neelsen, 2020

Figure 1 illustrates the hypothetical pathways mapping HFS reforms to health system outcomes through intermediate outputs and outcomes. More detailed pathway examples are provided in Appendix 1. A country with ‘predominant‐OOP HFS’ has its total health expenditure (THE) predominantly contributed through OOP, as identified via cluster analysis, and similarly for other classification categories (more on this in Section 3.1).

As shown in the above framework, governments may reform their HFSs to increase (pooled, pre‐paid) public revenues and public health expenditure. These efforts are in line with the recommendation of increasing public health expenditures in order to avoid that OOP expenditures increase impoverishment (Xu et al., 2003) and decrease utilization of health services (Qin et al., 2019). For example, assume a country with very high OOP health expenditures decides to subsidize completely all health services to under‐5 year old children and pregnant women. The pool of (fully) covered patients increases and more public revenues (payroll contributions and/or general taxes) are then raised to pay for those services. OOP expenditures of pregnant women and families with under‐5 year old children will be expected to decrease as services previously paid by OOP are now subsidized by the government via pre‐paid taxes: these families are now more protected against financial risk (related to OOP health expenditures). Government non‐contributory financing emerges as the largest contributor to THE, exemplifying what we call a transition (Fan & Savedoff, 2014a; World Health Organization, 2019) from OOP‐predominant to government‐predominant HFS. As in the health financing transition described in the literature (Fan & Savedoff, 2014a), THE per capita is likely to grow while government financing expenditure becomes predominant and OOP expenditure as percentage of THE decreases. Higher THE, especially via increased government financing and decreased OOP expenditure, would translate into more services offered to the population (Qin et al., 2019). Assuming there is demand for the services, the population will use more services than before, and, if those services are of sufficient quality, population health will improve (Moreno‐Serra & Smith, 2015). Similarly, THE may be spent more efficiently when it is pooled and pre‐paid: families paying for services OOP do not pool together their financial resources and pre‐pay for complex and efficient services, such as vaccination or public health campaigns, and neither can they share the risks of ill health across life‐stages (old‐young) or social strata (rich‐poor). Pre‐paid pooled expenditures in principle allow the government to deliver cost‐effective, preventative health services such as vaccinations, community health, and others, as well as to pool the risks of different individuals together (Moreno‐Serra & Smith, 2015). In both these cases (i.e., when THE increases and/or when THE is spent more efficiently), if pregnant women and families with children under‐5 who utilize public services belong to poorer population groups, health equity may also be positively impacted.

Contextual factors and unintended consequences are included in the conceptual framework. Contextual factors are to a certain extent not directly part of—and to a certain extent may be external to—HFS transitions (e.g., quality of governance, education or income per capita), and may modify the effect of HFSs on health system outcomes (Rajkumar & Swaroop, 2008). One example of an unintended consequence is that the introduction of SHI funded via payroll contributions SHI may drive lower formal employment (Savedoff, 2004; Wagstaff & Moreno‐Serra, 2009b; Yazbeck et al., 2020), which in turn may reduce the amount of revenues generated to fund the system and hence limit SHI coverage.

Figure 1 also shows the effects investigated by previous studies (blue lines), and the effects investigated in this paper (red lines), highlighting our contribution to the literature. An important clarification is required: as shown by the red arrow, we do *not* investigate the effect of an SHI reform in the way this has been done in (Wagstaff, 2009a; Wagstaff & Moreno‐Serra, 2009a), which would classify as “SHI” any country with SHI policies, laws and institutions. The effect we investigate is that of *health expenditure transitions*, from being OOP‐predominant to being government‐ or SHI‐predominant. Take for example, Ghana, which introduced the National Health Insurance Fund (NHIF, a form of SHI) in 2004. By 2017, OOP still accounted for the largest proportion of Ghana's total health expenditures. Hence, Ghana is classified in this paper as an OOP‐predominant HFS country, rather than a SHI country, despite the existence of SHI laws and institutions. Therefore, our analysis does not estimate the effect of the introduction of the SHI policy in Ghana. Similarly, Brazil's public health system was instituted by law in 1990, but its HFS transitioned from being OOP‐predominant to being government‐predominant only in the 2000–2017 period: our study measures the effect of Brazil's (and other countries') health financing transition out of OOP expenditure, rather than the introduction of laws to expand primary healthcare services. As these examples illustrate, our baseline estimates of the effects for SHI‐ and government financing‐predominant HFS should be interpreted as the effects of SHI or government financing policies that are successful in making a HFS transition from OOP predominance to SHI or government financing predominance. SHI or government financing policies that fail to do so would result in HFS being classified as OOP predominant HFS. The effect of increased SHI or government financing expenditure that does not translate into a change in predominant HFS is explored in models where we use SHI and government finance as % of THE as treatment variables (Appendix 6), instead of using SHI‐ and government financing‐predominant HFS dummies, and in models exploring within‐group changes in financing arrangements percentages (Appendix 4).

## 3 METHODS

### 3.1 Health financing system definition

Different health financing arrangements tend to coexist in a country's HFS. The three major health financing arrangements contributing to THE are government financing, social health insurance (SHI) and OOP. These financing arrangements account for 89% of THE on average across all countries, all years (2000–2017); the remainder is largely voluntary health insurance, which includes community‐based health insurance. Details regarding data sources is in Appendix 2, while detail about health financing arrangements are presented in Appendix 3 (Wagstaff, 2009b).

Government financing is universal in that it provides healthcare coverage to the population automatically based on residency or citizenship status, *without* requiring a direct contribution. Health services are pre‐paid, usually by general taxation, and there is usually a common pool for all residents/citizens. Predominantly government‐financed countries, whose public health systems are often referred to as “national health service”, are for example, UK, Italy, Spain, Australia, Canada, and Cuba. Publicly funded health insurance schemes that are entirely non‐contributory (e.g., Thailand Universal Coverage Scheme (Sumriddetchkajorn et al., 2019), or India Ayushman Bharat Pradhan Mantri Jan Aarogya Yojana) are also considered government financing. Due to data limitations, non‐contributory government financing arrangements that show features typical of health insurance schemes (e.g., provider and payer split, health insurance premiums or budgets paid by the government) cannot be separated from other non‐contributory government financing arrangements. SHI‐financing is also pre‐paid, but it differentiates itself from government financing by being *contributory*: a contribution has to be paid for a person/household to be able to receive healthcare coverage. Traditionally, the contribution is a deduction from the person's payroll. Individuals or households that do not contribute are not covered. In recent ‘extended’ forms, population groups that are usually identified as unable or ineligible for payroll or premium contributions are covered through government subsidies out of general tax revenues. In either of these cases, there may be different pools in the same country. Examples of SHI‐predominant countries are for example, Germany, France, Austria, Japan, Poland, and Turkey. Both government financing and SHI financing are heterogeneous and implementation differs by country. OOP financing is generally characterized by private citizens buying or paying for health services when needed, without any pre‐payment or risk pooling. Some government financing‐ and SHI‐predominant HFSs may have OOP co‐payments made by citizens/members: these fees are included in OOP expenditures. OOP‐predominant countries are for example, Armenia, Bangladesh, Mali, Ecuador, Liberia and India.

Previous studies have classified into the “SHI” group those countries with SHI laws, SHI institutions and/or earmarked payroll deductions (Wagstaff, 2009b; Wagstaff & Moreno‐Serra, 2009a) (i.e., the Bismarck model). All other countries were usually classified as “tax‐based” (i.e., the Beveridge model (van der Zee & Kroneman, 2007)). This approach arguably runs the risk of potentially having misclassified OOP‐predominant countries as tax‐based HFS (Armenia, Azerbaijan, Ukraine, Uzbekistan, Kyrgyz Republic). As shown in Table 1, A1, A2, A4, in all these countries, OOP expenditure is the main contributor to THE. In addition, we provide examples from other countries not included in (Wagstaff, 2009a; Wagstaff & Moreno‐Serra, 2009a).

**TABLE 1:** Comparison of countries' health financing system (HFS) classification across studies

Country	HFS classification in this paper*	HFS:SHI or tax‐based (Wagstaff, 2009b; Wagstaff & Moreno‐Serra, 2009a)*	SHI financing as % of THE	Government financing as % of THE	OOP expenditures as % of THE
*Liberia*	*OOP*	*Tax‐based*	0	31.74	45.51
Armenia	OOP	Tax‐based	0	14.18	84.35
Azerbaijan	OOP	Tax‐based	0	15.45	83.86
Kyrgyz Republic	OOP	Tax‐based	6.760	35.47	56.38
Ukraine	OOP	Tax‐based	0	44.64	52.32
Uzbekistan	OOP	Tax‐based	0	44.98	53.43
*Bolivia*	*Government*	*SHI*	30.08	39.92	25.08
*Indonesia*	*OOP*	*SHI*	22.65	26.46	34.61
*Ecuador*	*OOP*	*SHI*	24.18	29.37	39.40
*El Salvador*	*OOP*	*SHI*	24.7	24.65	29.20
*Nicaragua*	*OOP*	*SHI*	24.12	39.67	32.60
UK	Government	Tax based	0	78.80	15.96
Italy	Government	Tax‐based	0	73.71	23.49
France	SHI	SHI	78.05	5.326	9.384
Germany	SHI	SHI	78.05	6.308	12.67
Hungary	SHI	SHI	61.09	8.118	26.89

*Note*: *possible classifications: OOP‐, government‐ or SHI‐predominant,. As an example, we take 2017 data. Countries in italics were not included in (Wagstaff, 2009b; Wagstaff & Moreno‐Serra, 2009a), we classified them based on the rules used in those papers. The sum of OOP, government financing and SHI as % of THE may not equal 100% due to other health financing arrangements (e.g., voluntary private health insurance arrangements, non‐resident arrangements).

*Source*: Author elaboration.

One option is to use expenditure data to define HFS via arbitrary thresholds. However, arbitrary choices may also misclassify countries with no clearly predominant financing arrangement.

By contrast, a clustering approach provides a classification that has two main benefits: it is largely data‐driven and uses as input health expenditures, rather than more arbitrary classification mechanisms based on information, which would be hard to interpret or collect across all world countries. Using k‐means clustering (MacQueen, 1967), each country‐year combination is assigned to the HFS that has the closest mean values of government‐, SHI‐ and OOP‐expenditure as percentage of THE. More detail regarding the clustering procedure is provided in Appendix 3. In this approach, the arbitrary choices are limited to the input factors and the number of groups. For the input factors, OOP, SHI and government financing expenditure as % of THE are chosen because, together, they make 89% of total health expenditure in our sample. Other schemes (non‐profit institutions serving households (NPISH), voluntary health insurance) are below 5% as a % of THE, and in no country‐year observation are found to be the largest scheme. We choose to have three groups because in this way we can better address the research question (i.e., the effect of transitions from OOP to SHI and government financing HFSs), and because clustering optimization analyses (Makles, 2012) suggest that three groups is an optimal choice (see Appendix 3). The HFS variable generated by the analysis has three possible values: government‐, SHI‐ and OOP‐predominant HFS by country‐year. We use the word “predominant” because in all cases HFS are a mix of different health financing arrangements: while one arrangement is predominant, other arrangements coexist. In fact, another benefit of clustering is that it recognizes the mixed nature of HFS by considering data regarding all three major health financing arrangements when assigning country‐year observations to HFS groups. In this paper, a health financing transition is defined as a country's “switch” that lasts at least 2 years from an OOP‐predominant HFS to a SHI‐ or government‐predominant HFS.

As the definition of the predominant HFS by country‐year may affect our results, we explore the robustness of our main results to different ‘predominant HFS’ definitions. First, we define the predominant HFS using the highest value between government‐, SHI‐ and OOP‐expenditure as percentage of THE. Second, to address concerns that country‐year observations may be classified as OOP‐predominant while having OOP expenditures as % of THE below 40% (see Table 1), we use different thresholds to define OOP‐predominant HFS. In other words, we define a country‐year observation as OOP‐predominant only if OOP expenditures as percentage of THE is larger than a threshold *t*, for example, 50%, 45%, 40%, etc. Third, we add other health financing arrangements variables to the clustering procedure so that all health financing arrangements making up 100% of THE (i.e., NPISH as % of THE, voluntary health insurance as % of THE, enterprise schemes as % of THE, and rest of the world schemes as % of THE) are considered.

### 3.2 Empirical strategy: Fixed effects and specification tests

#### 3.2.1 Empirical strategy

The main specification is as follows:

(1) $$Y_{it}=\alpha +\rho_{1}{\mathrm{SHI}}_{it}+\rho_{2}{\mathrm{GOV}}_{it}+\gamma X_{it}+T_{t}+C_{i}+\varepsilon_{it}$$

Where Y represents an outcome of interest from Figure 1, in country i at time t. SHI and GOV are HFS dummies that take value 1 if the country‐year observation respectively belongs to the SHI‐predominant or government‐predominant HFS group, and 0 otherwise. OOP is the reference HFS. X is a vector of control variables. T represents time fixed effects (FE), and C country FE, which respectively control for cross‐country shocks and time‐invariant unobservable variables. Coefficients $\rho_{1}$ and $\rho_{2}$ can be interpreted as the within‐country effect on outcome Y of transitioning (i.e., switching) from OOP, the reference category, to SHI‐ and government‐predominant HFS, holding controls (detailed later) constant.

To investigate the question “how does context matter”, we augment our model by interacting SHI‐ and government‐predominant HFS dummies with several contextual factors (Equation 2), detailed later.

(2) $$Y_{it}=\alpha +\beta_{1}\left({\mathrm{SHI}}_{it}\times {\mathrm{CF}}_{it}\right)+\beta_{2}\left({GOV}_{it}\times {CF}_{it}\right)+{CF}_{it}+{\mathrm{SHI}}_{it}+{GOV}_{it}+X_{it}+T_{t}+C_{i}+\varepsilon_{it}$$

For the contextual factor analysis, we are interested in the interaction terms coefficients $\beta_{1}$ and $\beta_{2},$ which will be interpreted as ${CF}_{it}$ modification on the effect of ${SHI}_{it}$ and ${GOV}_{it}$ on outcome $Y_{it}$ by computing ${SHI}_{it}$ and ${GOV}_{it}$ at different values of ${CF}_{it}$.

The main model in Equation (2) is similar to a generalized difference‐in‐difference (DiD) estimator, with two reversible treatments, one reference group (OOP predominant group), and different treatment timing (i.e., a country can switch from the OOP group to SHI or GOV groups and vice versa at any t). DiD assumes a parallel trend: we therefore subject our results to tests of the DiD parallel trend assumption as done in (Wagstaff, 2009a; Wagstaff & Moreno‐Serra, 2009a).

#### 3.2.2 Specification tests

We conduct tests of the parallel trend assumption using random trend and differential trend models. In the random trend model, we relax the parallel trend assumption by adding country‐specific linear trends ($c_{i}t$), as shown in the following equation:

(3) $$Y_{it}=\alpha +\rho_{1}{\mathrm{SHI}}_{it}+\rho_{2}{GOV}_{it}+\gamma X_{it}+T_{t}+C_{i}+c_{i}t+\varepsilon_{it}$$

We estimate Equation (3) with and without country‐specific linear trends. We then test whether the SHI and GOV effects are different in FE models with country‐specific trends (FECS) and FE models without them (FE) (Clogg et al., 1995):

(4) $$Z=\frac{\rho_{1FECS}-\rho_{1FE}}{\sqrt{{SE\left(\rho_{1FECS}\right)}^{2}+{SE\left(\rho_{1FE}\right)}^{2}}}$$

This test allows using SEs clustered at country level. Non‐rejection of the tests in Equation (4) (2 tests per model, one for $\rho_{1}$ and one for $\rho_{2}$) would suggest that $\rho_{\mathrm{FECS}}$ and $\rho_{\mathrm{FE}}$ are not different, that $c_{i}t$ are not correlated with SHI or GOV, and that the parallel trend assumption (PTA) is consistent with our data. This can be seen intuitively: Equation (3) without $c_{i}t$ is equal to Equation (1).

The random trend model assumes that each country trend is linear and is not affected by SHI and GOV. These assumptions are likely to not hold in our case, as it is likely that SHI and GOV affect country trends. We therefore relax the parallel trend assumption using a differential trend model (Blundell & Costa Dias, 2009; Wagstaff, 2009a; Wagstaff & Moreno‐Serra, 2009a). The error term is now:

(5) $$\varepsilon_{it}=\left\{\begin{array}{c} C_{i}+k_{S}m_{t}+\varepsilon_{it}\;if\;\mathrm{SHI}=1\\ C_{i}+k_{G}m_{t}+\varepsilon_{it}\;if\;\mathrm{GOV}=1\\ C_{i}+k_{O}m_{t}+\varepsilon_{it}\;if\;\mathrm{SHI}=\mathrm{GOV}=0 \end{array}\right.$$

Where $m_{t}$ is an unobserved (differential) trend whose effect on the outcomes is different across SHI‐, government‐ and OOP‐predominant countries. This allows each HFS group trend to be non‐linear and modified by SHI and GOV, as shown in the following equation:

(6) $$Y_{it}=\alpha +\rho_{1}{\mathrm{SHI}}_{it}+{\left(k_{S}-k_{O}\right)\mathrm{SHI}}_{it}m_{t}+\rho_{2}{GOV}_{it}+{\left(k_{G}-k_{O}\right)GOV}_{it}m_{t}+\gamma X_{it}+k_{O}m_{t}+C_{i}+\varepsilon_{it}$$

Equation (6) can be estimated via fixed effects, with interactions between year dummies (first year dummy is excluded and used as reference) and treatment dummies:

(7) $$Y_{it}=\alpha +\rho_{1}{\mathrm{SHI}}_{it}+\sum_{t=2}^{T}\;\rho_{1t}{\mathrm{SHI}}_{it}{YEAR}_{t}+\rho_{2}{GOV}_{it}+\sum_{t=2}^{T}\;\rho_{2t}{GOV}_{it}{\mathrm{YEAR}}_{t}+\gamma X_{it}+{\beta_{t}\mathrm{YEAR}}_{t}+C_{i}+\varepsilon_{it}$$

The effect of each transition can be calculated as the average effect of SHI and GOV, respectively:

(8) $$\begin{array}{c} Mean\;\mathrm{SHI}\;impact=\rho_{1}+\sum_{t=2}^{T}\rho_{1t}/T-1\\ Mean\;GOV\;impact=\rho_{2}+\sum_{t=2}^{T}\rho_{2t}/T-1 \end{array}$$

As shown in (Wagstaff, 2009a; Wagstaff & Moreno‐Serra, 2009a) the PTA in the differential trend implies that $\left(k_{S}-k_{O}\right)=\left(k_{G}-k_{O}\right)=0$, which can be tested via the following nonlinear restriction, for $\rho_{1t}$ and $\rho_{2t}$:

(9) $$\frac{\left(k_{S}-k_{O}\right)\sum_{t}m_{t}}{k_{O}\sum_{t}m_{t}}=\frac{\sum_{t=2}^{T}\rho_{1t}}{\sum_{t=2}^{T}\beta_{t}}=0\;\frac{\left(k_{G}-k_{O}\right)\sum_{t}m_{t}}{k_{O}\sum_{t}m_{t}}=\frac{\sum_{t=2}^{T}\rho_{2t}}{\sum_{t=2}^{T}\beta_{t}}=0$$

Again, non‐rejection of these tests would suggest that the PTA is consistent with our data. This can be seen intuitively: Equation (6) reduces itself to Equation (1) when $\left(k_{S}-k_{O}\right)=\left(k_{G}-k_{O}\right)=0$

Reverse causality does remain a concern, as a country will likely increase SHI and government financing when population health is deteriorating (e.g., a health crisis such as Ebola or COVID‐19): we expect that reverse causality will bias the estimated coefficients for SHI and government HFSs downward for life expectancy, and upward for mortality and catastrophic health expenditure incidence. We run a test of reverse causality (in a Granger sense) used in the related literature (Gruber & Hanratty, 1995; Wagstaff, 2009a; Wagstaff & Moreno‐Serra, 2009a), noting that the test does not necessarily imply causality (Angrist a&nd Pischke, 2008a). We add to Equations (1), Equations (3) and (7) lead HFS variables (${SHI}_{i,t+1}$, ${GOV}_{i,t+1}$) that indicate whether the following year there will be a transition from OOP to SHI or government financing. A non‐zero coefficient would suggest that endogeneity is not appropriately addressed, while a zero coefficient would indicate the opposite.

Finally, the recent literature on country and time FE regressions has highlighted the problem (“negative weights”) that, in the context of heterogeneous treatment effects, the FE estimator is a weighted average of different effects, including the treatment effect of early versus late treatment adopter, and vice‐versa (Goodman‐Bacon, 2018). We therefore decompose $\rho_{1}$ and $\rho_{2}$ (Equation 1) to explore whether this issue is affecting our results, for countries for which the transition was staggered (i.e., they remained exposed to the HFS they transitioned to).

Stata 14 (StataCorp, 2015) has been used. Heteroskedastic‐ and within‐panel serial correlation‐robust SEs, clustered at the country level, are reported. A replication package is provided in the data availability statement.

## 4 DATA

We use annual data for the 2000–2017 period across a global sample of countries from different sources; due to data limitations, our main models include 124 countries. Sample construction details, variables definition, and source datasets are provided in Appendix 2.

### 4.1 Health financing data

The data on health expenditures (by financing arrangement) as percentages of THE, which is used for the cluster analysis, are from the WHO Global Health Expenditure Database (GHED), for the 2000–2017 period. WHO collects GHED data from countries using the System of Health Accounts (SHA) 2011 methodology (OECD et al., 2017). We use data under the “Health Care Financing Schemes” section, classification codes HF.1–4. In this paper, we use “arrangement” as a synonym of scheme, to avoid confusion with HFS (health financing system). If tax revenues are used to finance a SHI agency providing contributory SHI coverage, those revenues are “channelled via” SHI and are counted as SHI expenditure. Predominance can be read as “health expenditures channelled predominantly via a” non‐contributory government, contributory SHI, or OOP arrangement, based on clustering results. As noted in the literature (Rannan‐Eliya, 2008), OOP financing estimates suffer from potential data quality concerns. SHI as a health financing scheme comprises both compulsory public health insurance (96% of total SHI, across all countries, 2000–2017) and compulsory private health insurance (4% of total SHI financing, across all countries, 2000–2017).

### 4.2 Intermediate outcomes and health system outcomes

Intermediate outcomes comprise the immunization coverage index (i.e., the average of measles, DPT and hepatitis immunization rates) from World Bank World Development Indicators (WDI) (World Bank, 2019c), and (logged) THE per capita in current US$ from WHO GHED. Health status health system outcomes are life expectancy (LE), maternal mortality (MM), and under‐5 child mortality (U5M), also from WDI. Mortality outcomes have been logged, as done in the related literature. The World Bank Health Equity and Financial Protection indicators (HEFPI) dataset (World Bank, 2019a) has been used for the financial risk protection health system outcomes. Since there are many different measures of financial risk protection, the most commonly used (Wagstaff et al., 2018) has been chosen: catastrophic health expenditure incidence at the 10% level (CAT 10%). Health equity and the UHC index are not used as an outcome due to data limitations. Data for the UHC index was available only for 2010 within the data period of the analysis 2000–2017 from the Global Burden of Disease UHC dataset (Lozano et al., 2020).

### 4.3 Contextual factors: Control variables and interaction terms

We select control variables (contextual factors in our conceptual framework, Figure 1) that may confound the relationship between public health expenditure and health outcomes (Nakamura et al., 2016). The WDI dataset was used for (logged) GDP per capita (PPP, constant 2011 US$), education (primary school enrollment gross %), urbanization rate, % population with drinking water access, Gini index, and proportion of population above‐65 and below‐14 (Nakamura et al., 2016). The Worldwide Governance Indicators (WGI) dataset (World Bank, 2019b) was used to extract the control variables government effectiveness and corruption control. We do not control for THE, hospital beds and health workforce, as these factors would be on the causal pathway between HFS and health system outcomes (i.e., “bad controls” (Angrist & Pischke, 2008b)).

Contextual factors used as interaction terms in Equation (2) are often cited as “conditions required for” HFS to be successful (Yazbeck et al., 2020): (logged) GDP per capita, government effectiveness, corruption control, percentage of health revenues from payroll contributions (i.e., labour‐tax), informal sector size (informal workers as % of non‐agricultural jobs), and general government expenditure (GGE) as % of GDP.

## 5 RESULTS

This section is organized as follows: first, we show clustering results, then we present FE estimates, and, finally, tests and robustness checks including sub‐sample analysis (e.g., for LMICs specifically) are shown.

### 5.1 Clustering analysis results

**FIGURE 2:**
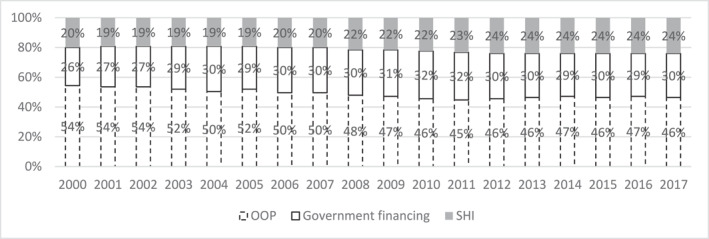
Proportion of 124 countries by HFS, year 2000 to year 2017. Source: Author elaboration. The graph represents the percentage of countries assigned to each predominant‐HFS per year

Figure 2 shows the results of the k‐means clustering analysis. In the 2000–2017 period, the proportion of predominantly‐OOP countries decreased (−8%), while SHI‐predominant and government predominant increased (+4% each). The clustering analysis confirms the health financing transition from OOP to public health expenditure (Fan and Savedoff, 2014b), that is, government and SHI HFS (World Health Organization, 2019).

Table 2 provides descriptive statistics for the three HFS groups. In SHI‐predominant country‐year observations, SHI channelled expenditure does *not* exceed 50% of THE, and there is slightly higher public health expenditure as a proportion of THE (i.e., sum of SHI and government financing as a % of THE) versus predominantly‐government financed systems. Table 2 suggests that selection into a HFS may not be random: SHI‐predominant observations show higher income, better health systems outcomes, higher THE, lower investments in primary health care (PHC) and lower informal sector size, versus other HFS′ groups. The OOP‐predominant HFS group is characterized, on average, by government financing being almost 30% of THE: in other words, OOP‐predominant systems are government financing systems with low public health expenditure.

**TABLE 2:** Means of main characteristics for full sample and across HFS clusters

Variable	Used as	Full sample	Predominant government HFS	Predominant OOP HFS	Predominant SHI HFS
*N (max)*		*2646*	*848*	*1282*	*516*
Life expectancy, at birth, years	Outcome	68.8	68.8	65.5	77.0
Under‐5 mortality, per 1000 live births	Outcome	44.3	39.4	62.1	8.3
Maternal mortality ratio, per 100,000 live births	Outcome	218.3	180.9	324.2	16.8
Catastrophic health expenditure, 10% threshold	Outcome	8.1	4.5	9.2	9.0
Immunization index	Outcome	85.3	89.1	79.7	93.0
Compulsory health insurance (SHI) as % of THE	Used to build HFS variable	16.0	2.7	7.3	59.3
Government financing as % of THE	Used to build HFS variable	36.1	62.6	28.6	11.0
Out‐of‐pocket (OOP) as % of THE	Used to build HFS variable	36.5	21.4	51.6	23.6
GDP per capita, PPP, current, international US$	Control and interaction term	15,394	21,662	7771	24,185
Corruption index	Control and interaction term	−0.10	0.293	−0.655	0.627
Government effectiveness	Control and interaction term	−0.05	0.242	−0.561	0.753
School enrollment, primary (% gross)	Control	102.0	103.5	100.7	102.6
% population using drinking water services	Control	83.0	83.5	76.6	98.0
% Population above 65 years old	Control	7.7	7.7	5.2	14.1
% Population below 14 years old	Control	29.6	28.8	34.7	18.4
Urbanization (% pop.)	Control	56.2	59.1	48.6	70.3
Gini index	Control	38.0	36.4	41.8	35.7
Health revenues from payroll contributions (%)	Interaction term	12.0	2.1	6.3	42.7
Informal sector size (% of non‐agricultural jobs)	Interaction term	57.38	48.40	65.95	32.68
GGE (% GDP)	Interaction term	30.31	34.47	23.90	39.38
GGHE (% GGE)	Intermediate outcome	9.7	10.4	7.6	13.7
THE (% GDP)	Intermediate outcome	6.1	6.0	5.5	7.9
THE per capita, PPP, current international US$	Intermediate outcome	1006	416	2046	1274
Primary health care expenditure, as a % of THE	Intermediate outcome	51.6	53.0	57.5	43.6

*Source*: Author elaboration, data: see Section 4.

**FIGURE 3:**
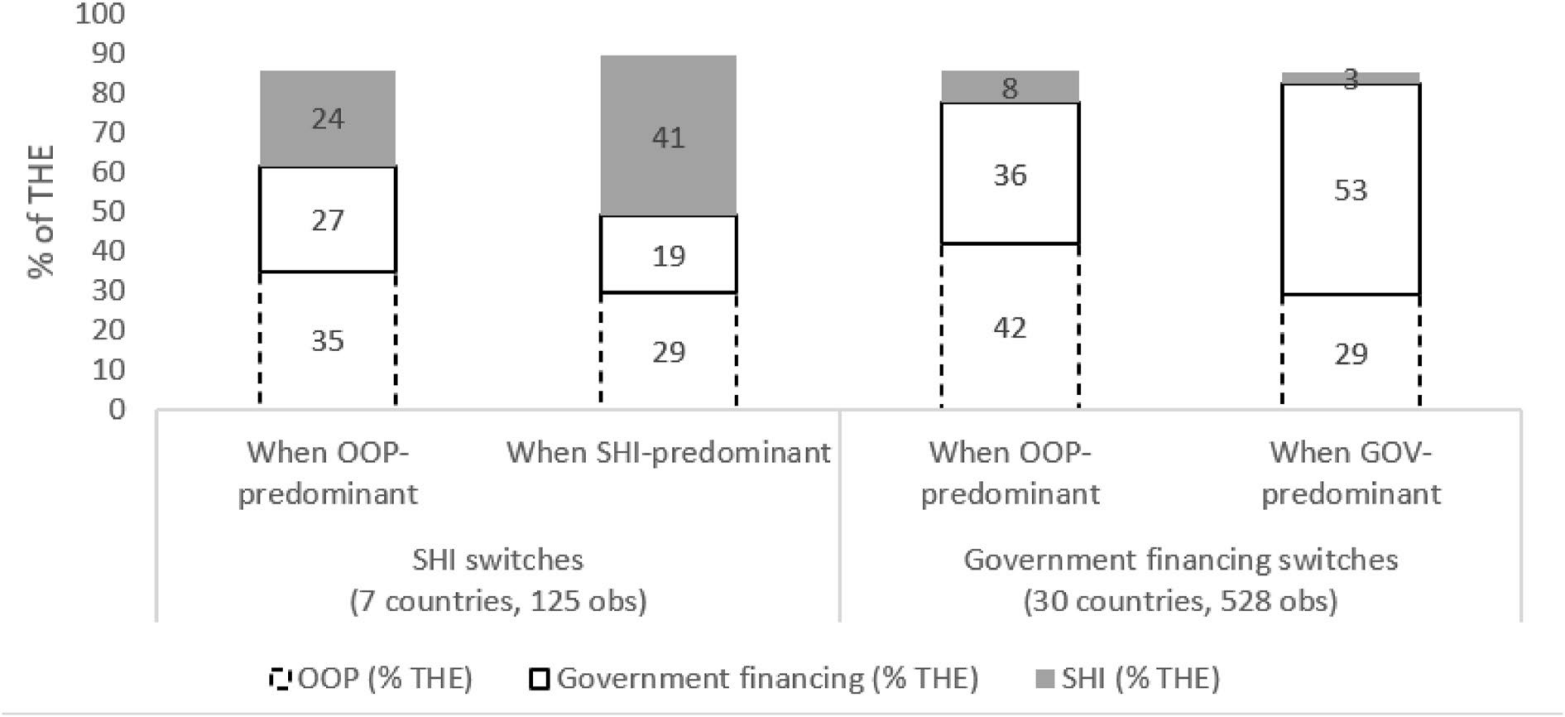
Average of OOP, SHI and government financing as % of THE, during health financing transitions. Source: Author elaboration. The figure shows SHI, OOP and government financing as % of THE for countries that switched from OOP‐ to SHI‐predominant and government financing‐predominant HFS. The sum of OOP, government financing and SHI as % of THE may not equal 100% due to other health financing arrangements (e.g., voluntary private health insurance, non‐resident arrangements)

**FIGURE 4:**
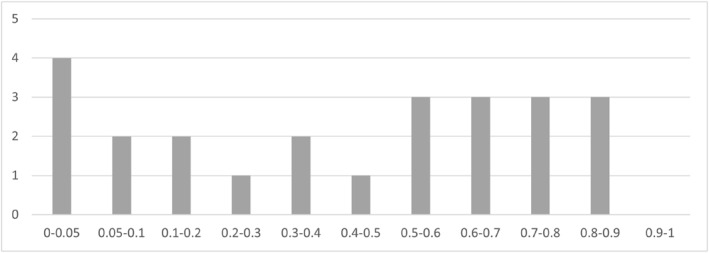
Histogram of parallel trend assumption specification tests *p*‐values. Source: Author elaboration. Histogram of *p*‐values resulting from PTA tests of the (Savedoff & Yazbeck, 2020) GOV and SHI dummy variables, across 2 specifications, random trend model PTA test (Equation 4) and differential trend model PTA test (Equation 9), all 6 outcomes (total of 24 tests)

Figure 3 focuses on the countries that switched from OOP to SHI or government financing HFS, or vice versa, in 2000–2017 (full list across 18 years in Appendix 3). Given that in FE regressions, within‐country variation is the focus (see 3.2.1), we note that seven countries switched from OOP to SHI, and 30 countries switched from OOP to government financing systems. SHI transitions show a lower decrease in OOP expenditures as % of THE (−6% percentage points), compared to government financing transitions (−13% percentage points). In both cases, the main public health expenditure arrangement increased significantly. In SHI transitions, not only OOP but also government financing did decrease (−8% of THE). For the government financing predominant HFS, the transition from OOP predominant to government financing predominant HFS is driven by growth in GGE as % of GDP (+6%) and growth in domestic health expenditure as % of GGE (+18%), which have finally resulted in a substantial increase in non‐contributory government financing as % of THE (Figure 4).

### 5.2 Regression results

Table 3 shows estimates from Equation (1). HFS coefficients in Table 3 represent the decrease/increase in the dependent variable (“outcome”) as a result of switching to a government‐ or SHI‐predominant HFS, from the reference OOP‐predominant system. HFS coefficients ρ for logged outcomes (THE per capita, U5M and MM) are interpreted as ${∆y\%=(e}^{\rho }-1)$.

**TABLE 3:** FE estimates for intermediate outcomes, health system outcomes

	Intermediate outcomes		HEALTH system outcomes			
	(1)	(2)	(3)	(4)	(5)	(6)
	Log THE per capita	Imm. Coverage	LE	Log U5M	Log MM	CAT 10%
	FE	FE	FE	FE	FE	FE
Government‐predominant	0.043	3.804	1.341**	−0.083**	−0.040	−3.256***
	(0.041)	(2.921)	(0.579)	(0.036)	(0.040)	(0.931)
SHI‐predominant	0.117***	−1.486	−0.128	0.051	0.034	6.467***
	(0.035)	(1.606)	(0.395)	(0.037)	(0.067)	(1.129)
Country FE	YES	YES	YES	YES	YES	YES
Year FE	YES	YES	YES	YES	YES	YES
Adjusted *R* ^2^	0.869	0.177	0.752	0.879	0.646	0.224
Observations	950	970	970	970	970	407
Number of countries	124	124	124	124	124	111

*Note*: FE estimates are the result of Equation (1). Robust SEs, clustered at country‐level, in parentheses. Details on HFS switches are detailed in Appendix 3. Full regression results including control variables are presented in Appendix 4. All models control for all variables listed as “control” in Table 2. *p*‐values for two‐sided *t*‐tests are reported as: ***p < 0.01, **p < 0.05, *p < 0.1.

*Source*: Author elaboration.

In terms of intermediate outcomes (as depicted in Figure 1), SHI transitions increase THE (column 1, +12.4%), while no such effect is visible for government financing transitions. FE estimates in column (Savedoff & Yazbeck, 2020) show that transitioning from a predominantly OOP to government‐ or SHI‐predominant HFS have effects that are not statistically different from zero on immunization coverage.

As for health system outcomes, transitioning from OOP‐ to government‐predominant HFS shows–for LE, U5M and CAT10%, respectively–rather strong evidence of an improvement (LE: +1.3 years, U5M: −8.7%, CAT10%: −3.3% points). Government‐predominant HFS transitions improve LE, U5M, and CAT10% (three out of four health system outcomes) significantly (*p* < 0.05) more so than SHI‐predominant HFS transitions. However, for CAT10%, the SHI lead tests–results not shown, see –suggest that SHI reverse causality may be a concern: since the SHI HFS lead is statistically different from zero, it appears that SHI transitions occur when CAT10% is high, and high CAT10% “anticipates” SHI transitions. No significant SHI transitions effects are found for maternal or under‐5 mortality.

One concern is that health financing mix heterogeneity *within* HFS groups may affect our results. For example, an increase in SHI‐financing as % of THE within the OOP predominant group may affect outcomes. To scrutinize this, we run FE regressions of government, SHI, and OOP expenditures as a percentage of THE on all outcomes within the government‐, SHI‐ and OOP‐predominant sub‐groups: in only six models out of 36, within‐group changes in financing arrangements show effects on outcomes different from zero (at 10% level) (see Appendix 4). In other words, within‐group heterogeneity in the percentage of expenditure channelled via different health financing arrangements has limited impact on outcomes.

### 5.3 How does context matter?

Estimates of Equation (2) using all six outcomes and six contextual factors (GDP per capita, informal sector size, proportion of health revenues from labor taxes, government expenditure as percentage of GDP, control of corruption, government effectiveness) are presented in Appendix 5. In seven of the 36 models estimated, at least one interaction term is significant (5% level), confirming empirically a non‐trivial role of contextual factors. We report on those significant estimates only.

The informal sector size is the contextual factor modifying the effect of HFS transitions in most cases: a one percentage point increase in informal sector size together with a transition to SHI‐predominant HFS increases U5M by 0.6%, and decreases immunization coverage by 0.2% points (the latter, when informal sector is beyond 65%). The same increase in informal sector size together with government financing HFS has a very similar effect on immunization coverage, but no effect on U5M. An increase in the log of GDP per capita improves the negative effect of SHI transitions on immunization coverage (+4.8% points), while better corruption control together with SHI‐predominant HFS transition delivered higher general government health expenditure as % of general government expenditure (+0.9% points in general government health expenditure per 1 point increase in the control of corruption index).

A percentage point increase in general government expenditure (as % of GDP) together with government‐predominant HFS transitions *decreases* general government health expenditure (% of general government expenditure), but the effect is very small (−0.08% points): possibly ministries of finance having large budgets tend to prioritize health sector funding slightly less as a proportion of total budget, when high absolute funding levels are considered sufficient. A one percentage point increase in health revenues coming from labor taxes together with SHI‐predominant transitions also *decreases* general government health expenditure (% of general government expenditure) (−0.12% points).

### 5.4 Specification tests and robustness checks

We present first the results of parallel trend assumption specification tests (Equations 4 and 9) which suggest that the parallel trend assumption is consistent with our data in the large majority of cases (∼75%), justifying the use of Equation (1) as our main specification. In the cases interested by potential parallel trend assumption rejections, we present random trend and differential trend model estimates (see Appendix 6, Panel G). These results do not change our conclusion.

**FIGURE 5:**
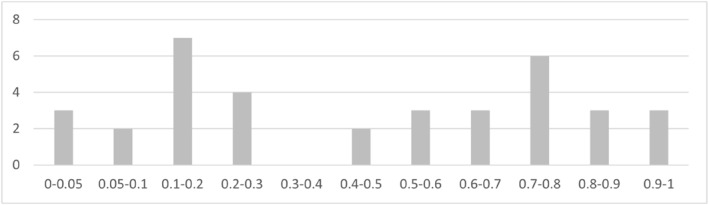
Histogram of reverse causality test *p*‐values. Source: Author elaboration. Histogram of *p*‐values of leads of GOV and SHI dummy variables regressed on 6 outcomes across 3 specifications: main DID specification, random trend model (Equation 3), and differential trend model PTA test (Equation 7) (total of 36 tests)

Second, we present in Figure 5 the results of the reverse causality tests (*p*‐values of GOV and SHI 1‐year leads in Equations (1), (3), and (7)): the vast majority of lead HFS (∼85%) are not significantly different from zero, suggesting that reverse causality is a rather limited issue across the vast majority of outcomes. However, the reverse causality (in a Granger sense) tests suggest that SHI transition occur when CAT 10% is particularly high, therefore the SHI coefficient for CAT 10% is likely affected by reverse causality.

Beyond specification tests, we subject our estimates to a series of robustness checks (Appendix 6). First, based on potentially very different contextual patterns in LMICs as compared to high‐income countries, we restrict the sample to LMICs. We also run sub‐group analyses restricting the sample to the high‐ and middle‐income countries, and to middle‐income countries only. Second, given concerns about public health data quality (Moreno‐Serra & Smith, 2015; Nakamura et al., 2016), we remove outliers (approx. 1% of the sample) using a non‐arbitrary methodology (Billor et al., 2000). Third, we use 1‐year lagged HFS independent variables as HFS effects on health system outcomes may not be contemporaneous. To explore the robustness of our main results to potentially lagged effects and reverse causality (in a Granger sense), we also implement visual event studies. Fourth, since general government expenditure as a percentage of GDP may limit the impact of HFS, we add it as a control variable. Fifth, we estimate Equation (1) using government and SHI as % of THE instead of HFS dummy variables (removing OOP expenditures as % of THE from the model due to collinearity issues, since the sum of all health financing arrangements is 100%). Sixth, we provide estimates of random trend and differential trend models in cases where the parallel trend assumption is rejected. Seventh, In the related literature, mortality outcomes have been either log‐transformed (Bokhari et al., 2007; Filmer & Pritchett, 1999; Rajkumar & Swaroop, 2008) or un‐transformed (Moreno‐Serra & Smith, 2015; Wagstaff, 2009a; Wagstaff & Moreno‐Serra, 2009a): to accommodate this alternative practice, in Panel H we use the natural units version of previously logged outcomes. Eighth, since the use of one of our control variables (Gini index) results in a loss of approximately half of total observations, we remove it to check for potential selection bias induced by missing observations. In addition, we remove other controls so that all countries in the dataset are included in the regression. Ninth, we add development assistance for health (as % of THE) to the list of control variables. Finally, we apply adjustments to all time‐varying controls that are related to HFS treatment (Zeldow & Hatfield, 2021).

Our baseline results are largely robust to the vast majority of the above‐mentioned specifications changes. Using random trend and differential models that relax the parallel trend assumption, the estimated coefficients on LE and CAT10% lose significance but show the same sign as the baseline coefficients. Adding unit‐specific linear trends (i.e., random trend model) would increase the importance of countries treated at the beginning and end of the panel (Goodman‐Bacon, 2018). For all other outcomes, baseline results are unaffected by relaxing the parallel trend assumption. In one specification (HFS percentages), the government HFS effect on LE loses significance. In the same specification, government financing improves CAT10% and U5M significantly more than SHI (not shown). Our results are particularly sensitive to this robustness check: while our clustering‐based HFS definition captures changes in the predominant HFS, HFS percentages capture changes in THE composition regardless of the predominant HFS. In other words, these results suggest that increasing SHI or government financing as a percentage of THE may not have a sizable impact on health outcomes, if the predominant HFS does not change.

Event studies confirm that government financing improves LE and U5M, in particular after 4–5 years, while SHI does not show improvements for any outcome. Using different definitions of HFS (i.e., using the highest value between government financing, SHI and OOP expenditures as a percentage of THE, and using OOP thresholds, as described in Section 3.1) does not substantially affect the main results either. In the “highest HFS value” specification, the coefficient for SHI effect on THE lose significance and the coefficient for SHI effect on logged MM shows a worsening, significant effect (+12.1% points, *p* < 0.01), suggesting possible health system outcomes worsening due to SHI transitions. The main results are also confirmed when adding all health financing arrangements variables in WHO GHED (i.e., NPISH as % of THE, enterprise schemes as % of THE, voluntary health insurance as % of THE), and when we add all health financing arrangement variables plus THE per capita as input variables to the clustering procedure: in either case, government financing HFS performs better than SHI HFS for all outcomes. Setting the number of clusters to four also shows that government financing predominant HFS perform better than predominant SHI HFS for U5M and CHE 10%, and never shows government financing being worse than SHI. Using additional health system outcomes (CHE 25%, impoverishment driven by OOP expenditures at the 1.90US$ and 3.20US$ poverty line, male and female adult mortality), and health system outcomes from different data sources (i.e., World Bank WDI “Maternal Mortality Ratio, National Estimates”; infant mortality and U5M from Demographic and Health Surveys), confirms that in most cases government financing predominant HFSs show better outcomes than SHI‐predominant HFSs.

Our baseline results are not affected by comparisons of late and early switchers (“negative weights”): the weight of $\rho_{1}$ and $\rho_{2}$ (Equation 1) driven by comparing late and early switchers outcomes is marginal for countries switching from OOP‐predominant to government financing HFS (weight 3%–5%) and to SHI‐predominant HFS (weight 1%–2%).

**FIGURE 6:**
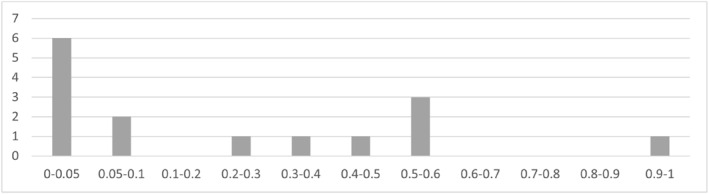
*p*‐values for difference between government financing and SHI coefficients. Source: Author elaboration. The Figure shows the histogram of *p*‐values for difference between government financing and SHI coefficients ($\rho_{1}-\rho_{2}=0$ in [1], [3], [7]). Whenever the *p*‐value is below 10%, there is at least suggestive evidence that government financing HFS transitions show better results than SHI HFS transitions. The *p*‐values are 15, resulting from three models (FE model, random trend model and differential trend model) times five health system outcomes (LE, U5M, MM, CAT 10%, immunization). THE is not considered as an outcome because larger health expenditure is desirable only if larger expenditure translates into more services coverage

Across our three main specifications (DiD FE, random trend, and differential trend models), government financing transitions improve (at the 10% level) outcomes more than SHI transitions in most cases (53% of the time, Figure 6). The effects of SHI transitions on health outcomes do not exceed that of government financing transitions for any of the outcomes. Estimates from robustness checks largely confirm these conclusions.

## 6 DISCUSSION

Achieving UHC is a widely shared health policy objective, and several countries are considering health financing systems (HFS) reforms (Yazbeck et al., 2020) to accelerate progress toward UHC. These HFS reforms seek to accelerate the health financing transition (Fan & Savedoff, 2014b; World Health Organization, 2019) from OOP‐predominant to public health expenditure (i.e., SHI‐ or government‐predominant expenditure as % of THE) predominant HFS. As policymakers face alternative health financing paths, it is important to understand what (if any) differences to health system outcomes they make.

Our main research objective has been to investigate the effect of transitions from OOP‐predominant to government‐ or SHI‐predominant HFSs on health system outcomes (i.e., health status, financial risk protection and utilization). Based on a conceptual framework for HFS transitions, we model HFS transitions from OOP‐predominant to SHI‐ and government‐predominant HFSs, assigning each country‐year observation to a predominant HFS using clustering—a machine learning approach. We estimate the effect of HFS transitions on intermediate and health system outcomes via FE regressions, controlling for time‐invariant as well as several contextual factors, while excluding potential “bad controls” (Angrist & Pischke, 2008b) on the causal pathway.

Transitions from OOP‐ to both government‐predominant and SHI‐predominant HFSs are both expected to deliver health system outcomes improvements via increased public health expenditure (see Section 2). However, we find that the effects of government‐predominant HFS transitions was more favourable than SHI‐predominant HFS transitions, for most outcomes. For the few outcomes where this was not the case, SHI and government‐predominant HFSs showed similar results. Hence, there is no outcome for which SHI transitions showed significantly better outcomes than government financing. These results are robust to most checks and tests.

Why do transitions to government financing appear to be superior to those to SHI? While we do not conduct a formal mediation analysis, we discuss several hypotheses on channels of influence, commenting on how the data may or may not support each possible channel.

The main difference between government and SHI financing is that SHI requires contributions made by or on behalf of the person accessing healthcare services. Despite recent cases of general taxation funding SHI expenditure (World Health Organization, 2019), SHI remains mostly financed by regular, typically wage‐related contributions (i.e., labor taxes, see Table 2): for many LMIC countries, this means that while formal workers are covered via compulsory contributions, for large parts of the population (i.e., informal workers) insurance coverage is voluntary (Barasa et al., 2021). SHI arrangements to cover the uninsured vary considerably across countries, and may generate pool fragmentation and pro‐rich bias (Barasa et al., 2021) (e.g., a pool with comprehensive benefit package for well‐off formal workers, and another one with a limited benefit package for the poor, the elderly, or an otherwise defined population group). Even when the non‐contributing poor or vulnerable are covered by subsidies, the informal non‐poor may be left out of affordable and quality options (Wagstaff, 2010). In our findings, informal sector size turns out indeed as the contextual factor with the biggest negative impact on the effects of HFS transitions.

SHI expansions may also come at higher costs and take longer time, compared to expansions of existing government financing mechanisms (see column (World Health Organization, 2020), Appendix 6). SHI requires institutional, technical and managerial capacity, and substantial investment to collect revenues and manage the provider‐payment system (Wagstaff, 2010). Limited regulatory capacity of purchasing institutions has been noted as a key issue (Wagstaff, 2010), and the time to develop capacity is not negligible: several countries in Western Europe took more than 70 years to reach UHC via SHI (Carrin & James, 2005). Expanding existing government financing arrangements would likely require less costs and time. The non‐healthcare‐related costs of SHI introductions or expansions may increase public health expenditure versus an OOP‐predominant‐system, with little improvements to healthcare coverage and finally health outcomes. SHI HFS have also traditionally focused on secondary/tertiary healthcare (Wagstaff, 2010) (suggested by Table 2, PHC expenditure descriptive statistics), which may be less efficient than PHC (Anderson et al., 2018). A full assessment of the relative performance of different types of HFS reforms would of course require a comparison of both the incremental costs and benefits of either HFS‐type–a challenge that is beyond the scope of this paper, and one that has hitherto not been met in the existing research (Kreif et al., 2021).

SHI transitions appear to not have succeeded in decreasing OOP expenditures as % of THE by as much as government financing transitions. SHI transitions decreased the reliance of THE on OOP expenditures, but they did so partially at the expense of non‐contributory government financing (see Figure 3). By contrast, government financing transitions did not result in a significant decrease in SHI financing (as % of THE), as illustrated by the experience of Moldova and Russia (see Appendix 7): increases in SHI expenditure (as % of THE) were accompanied by substantial decreases in government financing (as % of THE), less so in OOP (as % of THE), and a flattening of the U5M curve. At the same time, THE in both countries continued to grow.

Estimates using SHI and government financing as a % of THE (rather than predominant financing dummy variables) do not support the idea of SHI as a complementary arrangement either (Appendix 6, Panel F). Increases in SHI expenditure (% of THE) increased THE, but did not improve outcomes. This is compatible with the hypothesis that SHI for formal workers may result in pool fragmentation and pro‐rich health expenditure (Wagstaff, 2010), and that implementation costs are a reason for SHI's limited effects. Both these issues arise regardless of SHI being a complementary or a predominant HFS. Rather than introducing SHI as a complementary arrangement, favourable SHI features (e.g., provider‐purchaser split, explicit benefit packages entitlement, beneficiaries included in governance bodies, covering vulnerable groups via ad‐hoc interventions (Wagstaff, 2009a; Wagstaff, 2010; Wagstaff & Moreno‐Serra, 2009a)) could be included in existing government financing systems, and vice‐versa (e.g., via removing SHI link between contributions and services' access, making it de‐facto government financing).

Government‐financed systems may have undesirable features, too. While automatic universal coverage is a positive feature, benefit packages are often too ambitious, so that the “depth” of this coverage and the actual package of services delivered is often limited in LMICs (Wagstaff, 2013). Often, the purchaser‐provider split is missing, and when it is present, there is no joint decision‐making body, which includes purchaser(s), covered populations and providers. These arrangements can be implemented in government‐predominant HFS, but they are more typical of SHI‐predominant HFS. SHI‐predominant HFS could see a positive healthcare coverage effect from efficient purchaser‐provider systems: however, for such effect to materialize, a well‐functioning provider network is required. Assuming that a higher GDP per capita may mean better provider networks, the fact that SHI transitions have a more beneficial effect on immunization coverage when GDP per capita is higher (see Appendix 5 and Section 5.3) seem to support this idea. Similarly, a realistic and explicit benefit package, and the idea of entitlement provided by SHI, are seen as the main advantages of SHI (Yazbeck et al., 2020), and could be considered for inclusion in government financing systems.

Other contextual factors play a role, too. Perhaps counter‐intuitively, higher labour‐tax financing resulted in *decreases* in government health expenditure (in % of general government expenditure), for SHI‐predominant HFS transitions (see Appendix 5, column 36). Ministries of Finance may respond to higher SHI labour‐tax revenues by decreasing transfers from general tax revenues to health. While we find no evidence that a (proportional) increase in labour‐tax revenues modifies HFS effects on health, labour‐tax increases may increase informal sector size (Wagstaff, 2010; Wagstaff & Moreno‐Serra, 2009b), which we find worsen SHI effects on health (Appendix 5, columns 1–6). Since we do not investigate the HFS impact on labor outcomes, and research in LMICs on this topic is limited (Le et al., 2019), this is an area for further research.

Many countries are contemplating SHI reforms for different reasons (Savedoff & Yazbeck, 2020; Yazbeck et al., 2020): increasing financial autonomy and increased budgets for health via earmarked‐to‐health labor taxes, the political attraction of providing entitlements (usually to formal sector workers, which include civil servants), and considering SHI enrollment as the UHC coverage measure. With government financing, all citizens/residents are covered, and the issue is the depth of such coverage, which is difficult to measure, while with SHI there is the SHI coverage measure to report on as “progress toward UHC”. Further research could focus on other reasons driving a resurgence in SHI reforms.

Since concerns about reverse causality and the parallel trends assumption could not be entirely resolved, and our results were robust to most–but not all–different specifications and outcomes, we do not claim to have presented fully causal impact estimates. The limitations that are to be borne in mind when interpreting the findings include: first, we do not take into consideration more extensive HFS and health system heterogeneity due to data limitations. Other health system features (e.g., gatekeeping, different provider‐payment systems, pooling fragmentation, private‐public providers, provider networks, governance structures, etc.) may also affect health system outcomes, but data on a global scale does not exist to capture those. Second, we note that the sample comprises only seven largely middle‐income countries that transitioned from OOP to SHI predominant systems (as mentioned in Section 5.1). However, interactions of the HFS treatment variable with log GDP per capita show limited heterogeneity in the effect of HFSs due to changes in log GDP per capita (Appendix 5), suggesting that this might not be a major issue. Finally, we have not addressed formally “how” (e.g., via mediation analysis) or “for whom” different HFSs work (e.g., health equity), due to data limitations.

While bearing these caveats in mind, the policy implication of these findings is that policymakers considering SHI transitions to accelerate progress toward UHC should take these results as a call for caution. For LMIC policymakers facing the challenge of large informal sectors, re higher poverty rates, and often not‐well‐functioning provider networks, the odds of accelerating progress toward UHC via introduction or expansion of contributory SHI appear more contained, as noted in the recent literature (Barasa et al., 2021). Pursuing the road toward non‐contributory financing expansions to accelerate progress toward UHC would appear as the more promising avenue, based on our findings. But then again, one cannot exclude the possibility that SHI can be made to work well for health system outcomes, and we cannot present non‐contributory government financing as being unambiguously superior to contributory SHI in every situation (Savedoff, 2004; Wagstaff, 2010; Yazbeck et al., 2020). For both expansions, our contextual factors analysis findings suggest that SHI performs better when informal sector is smaller, GDP per capita is higher, and, to a lesser extent, when control of corruption is higher and labor tax financing is lower. Other contextual factors that may improve the effects of SHI transitions comprise higher wages, functioning provider networks, higher government technical, regulatory and financial capacity, and lower average household size (Hsiao et al., 2007). Information regarding these contextual factors, and their expected trend, can further strengthen decision‐making confidence regarding HFSs reforms. These policy implications and findings are also relevant for governmental and non‐governmental development partners supporting governments in moving toward UHC via technical and financial assistance.

## CONFLICT OF INTEREST

None of the authors has any conflict of interest to declare.

## ETHICS STATEMENT

We did not have to obtain ethical approval. All data used is secondary country‐level data from publicly available sources.

## Figures and Tables

**FIGURE A1:**
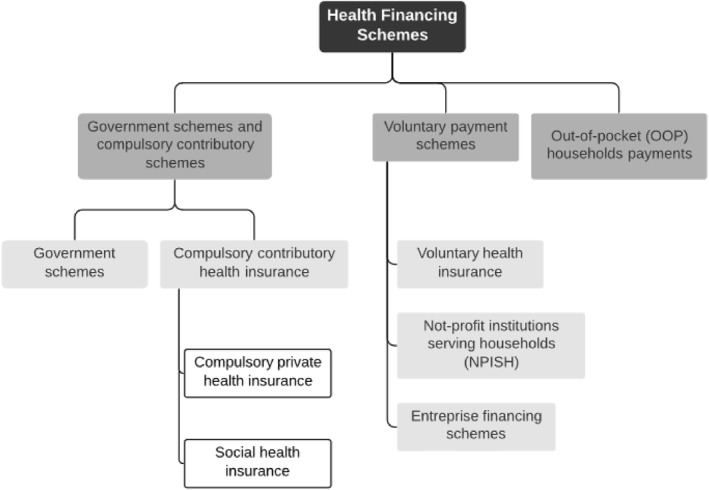
Health financing schemes definitions. Source: Author elaboration based on OECD, Eurostat, WHO, 2017, Chapter 7

**Figure:**
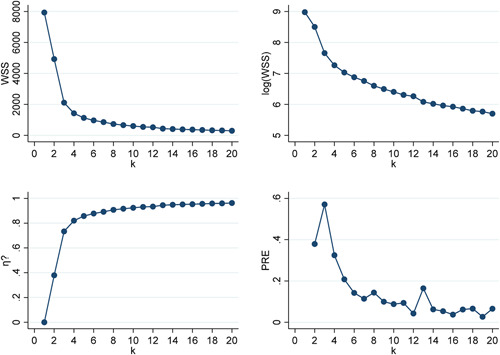
Source: Authors elaboration. The above figures are the results of the calculations described in the previous paragraph for WSS, ln(WSS), eta squared, PRE

**Figure:**
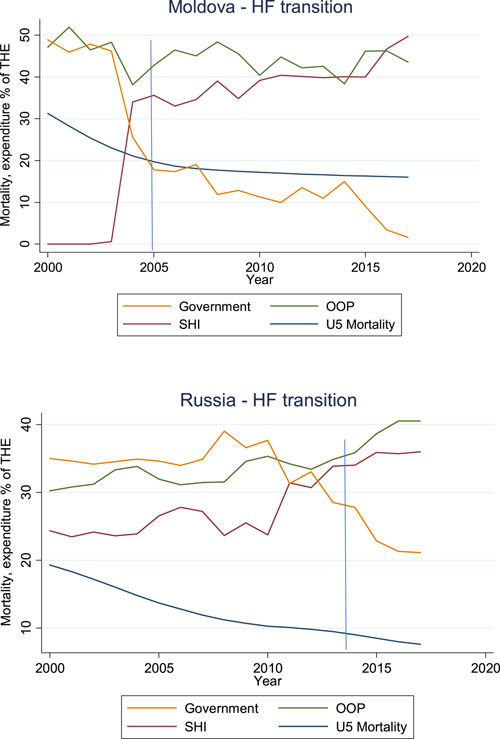
Source: Author elaboration. Descriptive data for selected variables, for Russia and Moldova, over time

## Data Availability

Datasets are detailed in the data section, and corresponding links are in the references section. Datasets and Stata do files are available upon request to the corresponding author, and a replication package with all datasets and do files is also available at the Open Science Framework repository https://osf.io/snczj/.

## APPENDIX 1. Differentiation across reforms of different health financing functions

The below figure helps clarify the idea that health financing reforms may impact health system outcomes through different pathways depending on the health financing function that is being reformed, as shown below.

## APPENDIX 2. Detail of variables and data construction

Sample construction:

**Table:**

Item	Number of countries from item	Number of countries (cumulative)
Total number of countries after merging all datasets and deleting countries not present in all datasets	183	183
Countries taken out because not all years present in a dataset	8 (Zimbabwe, Afghanistan, Yemen, Syria, Lybia, Iraq, South Sudan, Timor‐Leste)	175
Country missing outcome (life expectancy or maternal mortality) for at least 1 year	6 (Andorra, Dominica, Marshall Islands, Palau, San Marino, St. Kitts and Nevis)	169
Countries missing GHED data for at least 1 year	3 (Greece, Saudi Arabia, Albania)	166
Countries taken out because population <500.000 people in at least one period	18 (Bahamas, Barbados, Belize, Cabo Verde, Grenada, Iceland, Kiribati, Luxembourg, Maldives, Malta, Micronesia, Samoa, Sao Tome and Principe, Seychelles, Solomon Islands, St. Lucia, Suriname, Tonga, Vanuatu)	147
Countries missing control variables	23 (United Arab Emirates, Bahrain, Bosnia Herzegovina, Cuba, Djibouti, Eritrea, Gabon, Equatorial Guinea, Guyana, Haiti, Japan, Cambodia, Kuwait, Lebanon, Myanmar, Namibia, New Zealand, Oman, Papua New Guinea, Qatar, Singapore, Turkmenistan, Trinidad and Tobago)	124
Total countries in study		124

*Note*: The table describes the sample construction.

*Source*: Author elaboration.

We have taken out 18 small and island countries, given that governance, health systems and health financing for those countries present peculiarities when compared to other countries. In small and island countries, changes in predominant HFS are more frequent than other countries (0.72 changes per country for small and island countries, vs. 0.43 in other countries, in the 2000–2017 period), and there are transitions from SHI to government financing system (and vice‐versa) that are not seen in other countries, raising concerns about data quality and relevance in the context of a cross‐country analysis.

Baseline results change when these countries are included, and when the HFS is defined via clustering. However, the main conclusion that the effect of government financing on health system outcomes is more or as favourable as that of SHI transitions is maintained. We also include all countries and define the predominant HFS using the highest value among government financing, SHI and OOP expenditures as % of THE, showing that estimates are similar to our baseline results and that, again, the conclusion that government financing effect on health system outcomes is better or as good as SHI is confirmed. These checks are presented with other robustness checks in Appendix 6, Table A3, panels E and F.

Variable definitions and source:

**Table:**

Variable	Definition	Source
Government financing as % of THE	“Participation is automatic: For all citizens/residents; or a specific group of the population (e.g., the poor) defined by law/government regulation.”	WHO GHED (WHO, 2019), based on OECD SHA. Definition is quoted from OECD SHA (World Bank, 2019a).
SHI as % of THE	“Participation is mandatory: For all citizens/residents; or a specific group of the population defined by law/government regulation. In some cases, however, the enrollment requires actions to be taken by the eligible persons.”	
OOP expenditures as % of THE	“Participation is voluntary: Willingness to pay of the household.”	
Immunization coverage	Average of immunization coverage for measles, DPT and hepatitis	World Bank WDI
Life expectancy	Life expectancy at birth	World Bank WDI
Maternal mortality	Maternal mortality ratio, modeled estimate, per 100,000 live births	World Bank WDI
Under‐5 mortality	Under‐five mortality rate (death per 1000 live births)	World Bank WDI
Catastrophic health expenditure	Catastrophic health expenditure incidence (% population), at the 10% threshold	World Bank HEFPI
GDP per capita	GDP per capita, current, international US$, PPP	World Bank WDI
Primary school enrollment (gross, %)	Primary school enrollment (gross, % of population)	World Bank WDI
Urbanization rate	% Population in urban areas	World Bank WDI
Drinking water access	% Population with drinking water access	World Bank WDI
Demographics: population below 14	% population below 14	World Bank WDI
Demographics: population above 65	% population above 65	World Bank WDI
Government effectiveness	“Government effectiveness captures perceptions of the quality of public services, the quality of the civil service and the degree of its independence from political pressures, the quality of policy formulation and implementation, and the credibility of the government's commitment to such policies”	World Bank WGI, definition quoted from https://info.worldbank.org/governance/wgi/Home/downLoadFile?fileName=ge.pdf
Corruption control	“Control of corruption captures perceptions of the extent to which public power is exercised for private gain, including both petty and grand forms of corruption, as well as "capture” of the state by elites and private interests.”	World Bank WGI, definition quoted from: https://info.worldbank.org/governance/wgi/Home/downLoadFile?fileName=cc.pdf

## APPENDIX 3. Health financing arrangement details, and k‐means clustering

The health financing arrangement classifications (government financing, SHI and OOP) are not decided by the authors: they are defined by the System of Health Accounts (SHA), Health Financing Scheme (Chapter 7) standards, which form the basis for the WHO GHED. The reader is referred to the SHA manual for a detailed description of the health financing schemes mentioned and used in the paper.

Figure A1 clarifies the meaning of government‐financed, SHI‐financed and OOP‐financed. Government financing refers to “Government schemes”. SHI‐financing refers to Compulsory contributory health insurance schemes, which includes both SHI‐proper and private compulsory health insurance. We call it SHI‐financing to avoid confusion, since this is the usual name in the literature. Community based health insurance (not shown in the figure) is included under voluntary payment schemes. We also emphasize that in WHO GHED, what matters is the health financing scheme through which the monies are spent: if tax revenues are used to finance the country SHI agency providing contributory SHI, then such monies will be recorded under SHI (see p. 170 of the SHA 2011 manual (StataCorp, 2015)). However, if government pays premiums or finance the budget of an agency providing non‐contributory health insurance or services, those monies count as government financing. To avoid confusion, THE in this paper refers to “current health expenditure” in the GHED dataset. Finally, we note that external financing (i.e., development assistance for health) will be considered part of any given country's public financing scheme (i.e., SHI or government financing) if on‐budget. When off‐budget, external financing will largely be considered as financing via not‐for‐profit institutions serving households.

K‐means clustering (MacQueen, 1967) is an unsupervized machine learning technique described in many books. The k‐means cluster algorithm assigns each country‐year combination (i.e., each observation) to the cluster with the least squared Euclidean distance (other distances can be used), which is the cluster with the closest mean.

In this study, we choose to have three clusters as we expect to have country‐year combinations that belong to one of the following three groups: predominantly government‐financed, SHI‐financed or OOP financed. We choose that the variables used for clustering are government‐, SHI‐, and OOP‐financing as a % of THE. The algorithm starts by assigning random cluster “centroids” (a vector of three values, government schemes, SHI, and OOP, as a % of THE) and assigning each country‐year combination to the nearest cluster (i.e., the cluster with the least Euclidean distance). At this point, a new cluster centroid is calculated: it is a vector of the mean of the country‐year combinations assigned to the cluster. The process is repeated until the cluster centroids position does not change. Via this process, k‐means minimizes intra‐cluster variance: in other words, the sum of squared distances between each country‐year combination and the centroid of the assigned cluster is minimized.

K‐means clustering therefore considers, for each country‐year combination, the vector of government schemes, SHI, and OOP, each as a % of THE, and that k‐means is non‐arbitrary, except in the choice of the number of clusters, which we do based on theoretical reasoning about having 3 groups (government schemes, SHI, and OOP) and the variables used for clustering (government schemes, SHI, and OOP, each as a % of THE). One problem with this approach is that countries may move repeatedly and in short period of times in‐and‐out a certain group, therefore switches lasting only 1 year have been removed. We note that inclusion in a group is reversible: countries can go from SHI to OOP or OOP to government financing, and vice versa.

It might occur that a country‐year observation is classified, for example, in the government financing HFS group, even if OOP as % of THE is higher than government financing as % of THE. This is because each country‐year observation is a vector of three values: government financing as % of THE, OOP as % of THE, and SHI as % of THE. Each cluster can also be thought of as a vector of government financing, OOP, and SHI, as % of THE (called centroids), which are measured as the means of all the country‐year observations within that same cluster.

The k‐means clustering algorithm does not formally consider which one of the three values forming the country‐year observation vector (government financing as % of THE, OOP as % of THE, and SHI as % of THE) is the highest. Country‐year observations are classified into each group (government financing, OOP, SHI) based on the country‐year observation vector Euclidean squared distance to the cluster centroids vector. Therefore, a country‐year observation with a very high OOP as % of THE might be classified as “government‐financing” when its distance to the government financing cluster is shorter than the distance to the OOP cluster. However, the OOP, government‐financing, and SHI cluster will show, respectively, OOP, government financing, and SHI as % of THE as the highest value of the three. This is because the k‐means clustering algorithm minimizes distance within clusters' observations and maximizes distance across clusters.

The centroids of the final clusters are the average OOP as % of THE, SHI as % of THE, and government financing as % of THE for each cluster, which are shown in Table 2, Section 5.1.

These clustering concerns are substantially less relevant when we run the following robustness checks:

(1) We classify country‐year into HFS groups/clusters using the largest value between OOP, SHI and government financing as % of THE. In 229 country‐year observations (8% of total observations) the predominant HFS defined via clustering is different from the predominant HFS defined by the “largest % of THE” method. An example of this is shown below, for all countries, Year 2017.
(2) We use a minimum threshold of OOP as % of THE to classify country‐year observations in the OOP‐predominant group that is, a country‐year can only be classified as OOP‐predominant if the cluster procedure identifies it as OOP predominant and its OOP as % of THE is higher than a certain threshold (50%, 45%, 40%, etc.)

We note that data is usually standardized when variables used for clustering are on different scales (e.g., age and income). In our case, all clustering variables are percentage of THE, therefore no standardization is required.

Number of switches resulting from cluster analysis, with countries in brackets:

**Table:**

Switch	Full sample (in italic, countries with missing controls data)
OOP ↔ SHI	8 switches, 7 countries (Argentina 2, Bulgaria 1, China 1, Moldova 1, Russia 1, Uruguay 1, USA 1) 7 switches from OOP to SHI, 1 switch from SHI to OOP
OOP ↔ GOV	46 switches, 30 countries (Angola 2, Burundi 3, Bulgaria, 1 Bolivia 1, Brazil 1, DRC 2, Rep of Congo 2, *Djibouti 1*, Ethiopia 3, Gambia 1, *Gabon 3*, Guinea‐Bissau 1, *Guyana 2*, Jordan 2, Kazakhstan 1, Kenya 1, Sri Lanka 1, Latvia 1, Madagascar 1, Mongolia 1, Mauritius 1, Malaysia 1, Panama 1, Rwanda 1, Tanzania 3, *Trinidad & Tobago 1*, Ukraine 2, Venezuela 2, Zambia 2) 28 switches from OOP to GOV 18 switches from GOV to OOP
SHI ↔ GOV	0

*Note*: The table lists all the switches from OOP predominant HFS to SHI or GOV (government financing) predominant HFS.

*Source*: Authors' elaboration.

There are 26 cases (1% of all country‐year observations), for which the clustering analysis resulted in a 1‐year long HFS switch. In such cases, the previous year predominant HFS was used: Bolivia, 2001; Central African Republic, 2015; Republic of Congo, 2009; Djibouti, 2003; Ethiopia, 2004; Gambia, 2002; Guinea‐Bissau, 2002; Guyana, 2010; Jamaica, 2001; Kazakhstan, 2002; Kyrgyzstan, 2009; Laos, 2009; Lebanon, 2012; Madagascar, 2002; Madagascar, 2009; Madagascar, 2013; Moldova, 2005; Moldova, 2009; Mauritius, 2003; Mongolia, 2005; Panama, 2016; Trinidad and Tobago, 2014; Tunisia, 2013; Ukraine, 2011; Uzbekistan, 2014; Vietnam, 2001.

Regarding the choice of the number of clusters, we measure the within‐sum‐of‐squares (WSS), the ln (WSS), $\eta^{2}$ defined as $\eta^{2}=1-\frac{\mathrm{WSS}\left(k\right)}{\mathrm{WSS}\left(1\right)}$ and the proportional reduction of error coefficient (PRE), defined as $PRE\left(k\right)=\frac{\mathrm{WSS}\left(k-1\right)-\mathrm{WSS}\left(k\right)}{\mathrm{WSS}\left(k-1\right)}$. While for WSS, ln (WSS), and $\eta^{2}$ the optimal cluster number is seen where there is a kink in the curve, or when the decrease becomes smaller versus previous decreases, in the PRE methodology the optimal cluster number is identified when the PRE(k) value is large (vs. other PRE(k) values). The graphs suggest that the optimal number of clusters is three, in line with the number of clustering input variables (OOP, SHI and government financing as % of THE, making 89% of THE on average). This is also in line with the literature on HFS, which often suggested using SHI and government financing predominant HFS systems (Wagstaff & Moreno‐Serra, 2009), to which we have added OOP predominant HFSs. We also run a robustness check using four clusters instead of three, and find that the overall conclusion is not changed.

**Table:**

Country	Code	2000	2001	2002	2003	2004	2005	2006	2007	2008	2009	2010	2011	2012	2013	2014	2015	2016	2017
Algeria	DZA	GOV	GOV	GOV	GOV	GOV	GOV	GOV	GOV	GOV	GOV	GOV	GOV	GOV	GOV	GOV	GOV	OOP	OOP
Angola	AGO	GOV	OOP	OOP	OOP	OOP	OOP	GOV	GOV	GOV	GOV	GOV	GOV	GOV	GOV	GOV	GOV	GOV	GOV
Argentina	ARG	SHI	SHI	SHI	SHI	OOP	OOP	OOP	OOP	SHI	SHI	SHI	SHI	SHI	SHI	SHI	SHI	SHI	SHI
Armenia	ARM	OOP	OOP	OOP	OOP	OOP	OOP	OOP	OOP	OOP	OOP	OOP	OOP	OOP	OOP	OOP	OOP	OOP	OOP
Australia	AUS	GOV	GOV	GOV	GOV	GOV	GOV	GOV	GOV	GOV	GOV	GOV	GOV	GOV	GOV	GOV	GOV	GOV	GOV
Austria	AUT	SHI	SHI	SHI	SHI	SHI	SHI	SHI	SHI	SHI	SHI	SHI	SHI	SHI	SHI	SHI	SHI	SHI	SHI
Azerbaijan	AZE	OOP	OOP	OOP	OOP	OOP	OOP	OOP	OOP	OOP	OOP	OOP	OOP	OOP	OOP	OOP	OOP	OOP	OOP
Bahrain	BHR	GOV	GOV	GOV	GOV	GOV	GOV	GOV	GOV	GOV	GOV	GOV	GOV	GOV	GOV	GOV	GOV	GOV	GOV
Bangladesh	BGD	OOP	OOP	OOP	OOP	OOP	OOP	OOP	OOP	OOP	OOP	OOP	OOP	OOP	OOP	OOP	OOP	OOP	OOP
Belarus	BLR	GOV	GOV	GOV	GOV	GOV	GOV	GOV	GOV	GOV	GOV	GOV	GOV	GOV	GOV	GOV	GOV	GOV	GOV
Belgium	BEL	SHI	SHI	SHI	SHI	SHI	SHI	SHI	SHI	SHI	SHI	SHI	SHI	SHI	SHI	SHI	SHI	SHI	SHI
Benin	BEN	OOP	OOP	OOP	OOP	OOP	OOP	OOP	OOP	OOP	OOP	OOP	OOP	OOP	OOP	OOP	OOP	OOP	OOP
Bhutan	BTN	GOV	GOV	GOV	GOV	GOV	GOV	GOV	GOV	GOV	GOV	GOV	GOV	GOV	GOV	GOV	GOV	GOV	GOV
Bolivia	BOL	OOP	OOP	OOP	OOP	OOP	OOP	OOP	OOP	OOP	OOP	OOP	OOP	OOP	OOP	OOP	OOP	OOP	GOV
Bosnia and Herzegovina	BIH	SHI	SHI	SHI	SHI	SHI	SHI	SHI	SHI	SHI	SHI	SHI	SHI	SHI	SHI	SHI	SHI	SHI	SHI
Botswana	BWA	GOV	GOV	GOV	GOV	GOV	GOV	GOV	GOV	GOV	GOV	GOV	GOV	GOV	GOV	GOV	GOV	GOV	GOV
Brazil	BRA	OOP	OOP	OOP	OOP	OOP	OOP	OOP	GOV	GOV	GOV	GOV	GOV	GOV	GOV	GOV	GOV	GOV	GOV
Bulgaria	BGR	GOV	OOP	OOP	OOP	OOP	OOP	SHI	SHI	SHI	SHI	SHI	SHI	SHI	SHI	SHI	SHI	SHI	SHI
Burkina Faso	BFA	GOV	GOV	GOV	GOV	GOV	GOV	GOV	GOV	GOV	GOV	GOV	GOV	GOV	GOV	GOV	GOV	GOV	GOV
Burundi	BDI	OOP	OOP	OOP	OOP	OOP	OOP	OOP	OOP	OOP	OOP	GOV	GOV	GOV	GOV	OOP	OOP	GOV	GOV
Cambodia	KHM	OOP	OOP	OOP	OOP	OOP	OOP	OOP	OOP	OOP	OOP	OOP	OOP	OOP	OOP	OOP	OOP	OOP	OOP
Cameroon	CMR	OOP	OOP	OOP	OOP	OOP	OOP	OOP	OOP	OOP	OOP	OOP	OOP	OOP	OOP	OOP	OOP	OOP	OOP
Canada	CAN	GOV	GOV	GOV	GOV	GOV	GOV	GOV	GOV	GOV	GOV	GOV	GOV	GOV	GOV	GOV	GOV	GOV	GOV
Central African Republic	CAF	OOP	OOP	OOP	OOP	OOP	OOP	OOP	OOP	OOP	OOP	OOP	OOP	OOP	OOP	OOP	OOP	OOP	OOP
Chad	TCD	OOP	OOP	OOP	OOP	OOP	OOP	OOP	OOP	OOP	OOP	OOP	OOP	OOP	OOP	OOP	OOP	OOP	OOP
Chile	CHL	SHI	SHI	SHI	SHI	SHI	SHI	SHI	SHI	SHI	SHI	SHI	SHI	SHI	SHI	SHI	SHI	SHI	SHI
China	CHN	OOP	OOP	OOP	OOP	OOP	OOP	OOP	OOP	OOP	OOP	OOP	OOP	SHI	SHI	SHI	SHI	SHI	SHI
Colombia	COL	SHI	SHI	SHI	SHI	SHI	SHI	SHI	SHI	SHI	SHI	SHI	SHI	SHI	SHI	SHI	SHI	SHI	SHI
Comoros	COM	OOP	OOP	OOP	OOP	OOP	OOP	OOP	OOP	OOP	OOP	OOP	OOP	OOP	OOP	OOP	OOP	OOP	OOP
Congo, Dem. Rep.	COD	OOP	OOP	OOP	OOP	OOP	OOP	OOP	OOP	OOP	OOP	GOV	GOV	OOP	OOP	OOP	OOP	OOP	OOP
Congo, Rep.	COG	OOP	OOP	OOP	OOP	OOP	OOP	OOP	OOP	GOV	GOV	GOV	GOV	GOV	GOV	GOV	GOV	OOP	OOP
Costa Rica	CRI	SHI	SHI	SHI	SHI	SHI	SHI	SHI	SHI	SHI	SHI	SHI	SHI	SHI	SHI	SHI	SHI	SHI	SHI
Cote d'Ivoire	CIV	OOP	OOP	OOP	OOP	OOP	OOP	OOP	OOP	OOP	OOP	OOP	OOP	OOP	OOP	OOP	OOP	OOP	OOP
Croatia	HRV	SHI	SHI	SHI	SHI	SHI	SHI	SHI	SHI	SHI	SHI	SHI	SHI	SHI	SHI	SHI	SHI	SHI	SHI
Cuba	CUB	GOV	GOV	GOV	GOV	GOV	GOV	GOV	GOV	GOV	GOV	GOV	GOV	GOV	GOV	GOV	GOV	GOV	GOV
Cyprus	CYP	OOP	OOP	OOP	OOP	OOP	OOP	OOP	OOP	OOP	OOP	OOP	OOP	OOP	OOP	OOP	OOP	OOP	OOP
Czech Republic	CZE	SHI	SHI	SHI	SHI	SHI	SHI	SHI	SHI	SHI	SHI	SHI	SHI	SHI	SHI	SHI	SHI	SHI	SHI
Denmark	DNK	GOV	GOV	GOV	GOV	GOV	GOV	GOV	GOV	GOV	GOV	GOV	GOV	GOV	GOV	GOV	GOV	GOV	GOV
Djibouti	DJI	OOP	OOP	OOP	OOP	OOP	OOP	GOV	GOV	GOV	GOV	GOV	GOV	GOV	GOV	GOV	GOV	GOV	GOV
Dominican Republic	DOM	OOP	OOP	OOP	OOP	OOP	OOP	OOP	OOP	OOP	OOP	OOP	OOP	OOP	OOP	OOP	OOP	OOP	OOP
Ecuador	ECU	OOP	OOP	OOP	OOP	OOP	OOP	OOP	OOP	OOP	OOP	OOP	OOP	OOP	OOP	OOP	OOP	OOP	OOP
Egypt, Arab Rep.	EGY	OOP	OOP	OOP	OOP	OOP	OOP	OOP	OOP	OOP	OOP	OOP	OOP	OOP	OOP	OOP	OOP	OOP	OOP
El Salvador	SLV	OOP	OOP	OOP	OOP	OOP	OOP	OOP	OOP	OOP	OOP	OOP	OOP	OOP	OOP	OOP	OOP	OOP	OOP
Equatorial Guinea	GNQ	OOP	OOP	OOP	OOP	OOP	OOP	OOP	OOP	OOP	OOP	OOP	OOP	OOP	OOP	OOP	OOP	OOP	OOP
Eritrea	ERI	OOP	OOP	OOP	OOP	OOP	OOP	OOP	OOP	OOP	OOP	OOP	OOP	OOP	OOP	OOP	OOP	OOP	OOP
Estonia	EST	SHI	SHI	SHI	SHI	SHI	SHI	SHI	SHI	SHI	SHI	SHI	SHI	SHI	SHI	SHI	SHI	SHI	SHI
Eswatini	SWZ	GOV	GOV	GOV	GOV	GOV	GOV	GOV	GOV	GOV	GOV	GOV	GOV	GOV	GOV	GOV	GOV	GOV	GOV
Ethiopia	ETH	OOP	GOV	GOV	GOV	GOV	GOV	OOP	OOP	OOP	OOP	OOP	OOP	OOP	OOP	OOP	OOP	OOP	GOV
Fiji	FJI	GOV	GOV	GOV	GOV	GOV	GOV	GOV	GOV	GOV	GOV	GOV	GOV	GOV	GOV	GOV	GOV	GOV	GOV
Finland	FIN	GOV	GOV	GOV	GOV	GOV	GOV	GOV	GOV	GOV	GOV	GOV	GOV	GOV	GOV	GOV	GOV	GOV	GOV
France	FRA	SHI	SHI	SHI	SHI	SHI	SHI	SHI	SHI	SHI	SHI	SHI	SHI	SHI	SHI	SHI	SHI	SHI	SHI
Gabon	GAB	OOP	OOP	OOP	OOP	OOP	OOP	OOP	OOP	OOP	OOP	GOV	GOV	GOV	GOV	OOP	OOP	GOV	GOV
Gambia, the	GMB	OOP	GOV	GOV	GOV	GOV	GOV	GOV	GOV	GOV	GOV	GOV	GOV	GOV	GOV	GOV	GOV	GOV	GOV
Georgia	GEO	OOP	OOP	OOP	OOP	OOP	OOP	OOP	OOP	OOP	OOP	OOP	OOP	OOP	OOP	OOP	OOP	OOP	OOP
Germany	DEU	SHI	SHI	SHI	SHI	SHI	SHI	SHI	SHI	SHI	SHI	SHI	SHI	SHI	SHI	SHI	SHI	SHI	SHI
Ghana	GHA	OOP	OOP	OOP	OOP	OOP	OOP	OOP	OOP	OOP	OOP	OOP	OOP	OOP	OOP	OOP	OOP	OOP	OOP
Guatemala	GTM	OOP	OOP	OOP	OOP	OOP	OOP	OOP	OOP	OOP	OOP	OOP	OOP	OOP	OOP	OOP	OOP	OOP	OOP
Guinea	GIN	OOP	OOP	OOP	OOP	OOP	OOP	OOP	OOP	OOP	OOP	OOP	OOP	OOP	OOP	OOP	OOP	OOP	OOP
Guinea‐Bissau	GNB	GOV	GOV	GOV	GOV	GOV	GOV	GOV	GOV	GOV	GOV	GOV	GOV	OOP	OOP	OOP	OOP	OOP	OOP
Guyana	GUY	GOV	GOV	GOV	GOV	GOV	GOV	OOP	OOP	OOP	GOV	GOV	GOV	GOV	GOV	GOV	GOV	GOV	GOV
Haiti	HTI	OOP	OOP	OOP	OOP	OOP	OOP	OOP	OOP	OOP	OOP	OOP	OOP	OOP	OOP	OOP	OOP	OOP	OOP
Honduras	HND	OOP	OOP	OOP	OOP	OOP	OOP	OOP	OOP	OOP	OOP	OOP	OOP	OOP	OOP	OOP	OOP	OOP	OOP
Hungary	HUN	SHI	SHI	SHI	SHI	SHI	SHI	SHI	SHI	SHI	SHI	SHI	SHI	SHI	SHI	SHI	SHI	SHI	SHI
India	IND	OOP	OOP	OOP	OOP	OOP	OOP	OOP	OOP	OOP	OOP	OOP	OOP	OOP	OOP	OOP	OOP	OOP	OOP
Indonesia	IDN	OOP	OOP	OOP	OOP	OOP	OOP	OOP	OOP	OOP	OOP	OOP	OOP	OOP	OOP	OOP	OOP	OOP	OOP
Iran, Islamic Rep.	IRN	OOP	OOP	OOP	OOP	OOP	OOP	OOP	OOP	OOP	OOP	OOP	OOP	OOP	OOP	OOP	OOP	OOP	OOP
Ireland	IRL	GOV	GOV	GOV	GOV	GOV	GOV	GOV	GOV	GOV	GOV	GOV	GOV	GOV	GOV	GOV	GOV	GOV	GOV
Israel	ISR	SHI	SHI	SHI	SHI	SHI	SHI	SHI	SHI	SHI	SHI	SHI	SHI	SHI	SHI	SHI	SHI	SHI	SHI
Italy	ITA	GOV	GOV	GOV	GOV	GOV	GOV	GOV	GOV	GOV	GOV	GOV	GOV	GOV	GOV	GOV	GOV	GOV	GOV
Jamaica	JAM	GOV	GOV	GOV	GOV	GOV	GOV	GOV	GOV	GOV	GOV	GOV	GOV	GOV	GOV	GOV	GOV	GOV	GOV
Japan	JPN	SHI	SHI	SHI	SHI	SHI	SHI	SHI	SHI	SHI	SHI	SHI	SHI	SHI	SHI	SHI	SHI	SHI	SHI
Jordan	JOR	OOP	OOP	OOP	OOP	OOP	OOP	OOP	GOV	GOV	GOV	GOV	GOV	GOV	GOV	GOV	GOV	GOV	OOP
Kazakhstan	KAZ	OOP	GOV	GOV	GOV	GOV	GOV	GOV	GOV	GOV	GOV	GOV	GOV	GOV	GOV	GOV	GOV	GOV	GOV
Kenya	KEN	OOP	OOP	OOP	OOP	OOP	OOP	OOP	OOP	OOP	OOP	OOP	OOP	OOP	OOP	OOP	GOV	GOV	GOV
Korea, Rep.	KOR	SHI	SHI	SHI	SHI	SHI	SHI	SHI	SHI	SHI	SHI	SHI	SHI	SHI	SHI	SHI	SHI	SHI	SHI
Kuwait	KWT	GOV	GOV	GOV	GOV	GOV	GOV	GOV	GOV	GOV	GOV	GOV	GOV	GOV	GOV	GOV	GOV	GOV	GOV
Kyrgyz Republic	KGZ	OOP	OOP	OOP	OOP	OOP	OOP	OOP	OOP	OOP	OOP	OOP	OOP	OOP	OOP	OOP	OOP	OOP	OOP
Lao PDR	LAO	OOP	OOP	OOP	OOP	OOP	OOP	OOP	OOP	OOP	OOP	OOP	OOP	OOP	OOP	OOP	OOP	OOP	OOP
Latvia	LVA	OOP	OOP	OOP	OOP	GOV	GOV	GOV	GOV	GOV	GOV	GOV	GOV	GOV	GOV	GOV	GOV	GOV	GOV
Lebanon	LBN	OOP	OOP	OOP	OOP	OOP	OOP	OOP	OOP	OOP	OOP	OOP	OOP	OOP	OOP	OOP	OOP	OOP	OOP
Lesotho	LSO	GOV	GOV	GOV	GOV	GOV	GOV	GOV	GOV	GOV	GOV	GOV	GOV	GOV	GOV	GOV	GOV	GOV	GOV
Liberia	LBR	OOP	OOP	OOP	OOP	OOP	OOP	OOP	OOP	OOP	OOP	OOP	OOP	OOP	OOP	OOP	OOP	OOP	OOP
Lithuania	LTU	SHI	SHI	SHI	SHI	SHI	SHI	SHI	SHI	SHI	SHI	SHI	SHI	SHI	SHI	SHI	SHI	SHI	SHI
Madagascar	MDG	OOP	GOV	GOV	GOV	GOV	GOV	GOV	GOV	GOV	GOV	GOV	GOV	GOV	GOV	GOV	GOV	GOV	GOV
Malawi	MWI	GOV	GOV	GOV	GOV	GOV	GOV	GOV	GOV	GOV	GOV	GOV	GOV	GOV	GOV	GOV	GOV	GOV	GOV
Malaysia	MYS	OOP	GOV	GOV	GOV	GOV	GOV	GOV	GOV	GOV	GOV	GOV	GOV	GOV	GOV	GOV	GOV	GOV	GOV
Mali	MLI	OOP	OOP	OOP	OOP	OOP	OOP	OOP	OOP	OOP	OOP	OOP	OOP	OOP	OOP	OOP	OOP	OOP	OOP
Mauritania	MRT	OOP	OOP	OOP	OOP	OOP	OOP	OOP	OOP	OOP	OOP	OOP	OOP	OOP	OOP	OOP	OOP	OOP	OOP
Mauritius	MUS	GOV	GOV	GOV	GOV	GOV	OOP	OOP	OOP	OOP	OOP	OOP	OOP	OOP	OOP	OOP	OOP	OOP	OOP
Mexico	MEX	OOP	OOP	OOP	OOP	OOP	OOP	OOP	OOP	OOP	OOP	OOP	OOP	OOP	OOP	OOP	OOP	OOP	OOP
Moldova	MDA	OOP	OOP	OOP	OOP	OOP	OOP	OOP	OOP	SHI	SHI	SHI	SHI	SHI	SHI	SHI	SHI	SHI	SHI
Mongolia	MNG	GOV	GOV	GOV	GOV	GOV	GOV	GOV	GOV	GOV	GOV	GOV	GOV	GOV	OOP	OOP	OOP	OOP	OOP
Morocco	MAR	OOP	OOP	OOP	OOP	OOP	OOP	OOP	OOP	OOP	OOP	OOP	OOP	OOP	OOP	OOP	OOP	OOP	OOP
Mozambique	MOZ	GOV	GOV	GOV	GOV	GOV	GOV	GOV	GOV	GOV	GOV	GOV	GOV	GOV	GOV	GOV	GOV	GOV	GOV
Myanmar	MMR	OOP	OOP	OOP	OOP	OOP	OOP	OOP	OOP	OOP	OOP	OOP	OOP	OOP	OOP	OOP	OOP	OOP	OOP
Namibia	NAM	GOV	GOV	GOV	GOV	GOV	GOV	GOV	GOV	GOV	GOV	GOV	GOV	GOV	GOV	GOV	GOV	GOV	GOV
Nepal	NPL	OOP	OOP	OOP	OOP	OOP	OOP	OOP	OOP	OOP	OOP	OOP	OOP	OOP	OOP	OOP	OOP	OOP	OOP
Netherlands	NLD	SHI	SHI	SHI	SHI	SHI	SHI	SHI	SHI	SHI	SHI	SHI	SHI	SHI	SHI	SHI	SHI	SHI	SHI
New Zealand	NZL	GOV	GOV	GOV	GOV	GOV	GOV	GOV	GOV	GOV	GOV	GOV	GOV	GOV	GOV	GOV	GOV	GOV	GOV
Nicaragua	NIC	OOP	OOP	OOP	OOP	OOP	OOP	OOP	OOP	OOP	OOP	OOP	OOP	OOP	OOP	OOP	OOP	OOP	OOP
Niger	NER	OOP	OOP	OOP	OOP	OOP	OOP	OOP	OOP	OOP	OOP	OOP	OOP	OOP	OOP	OOP	OOP	OOP	OOP
Nigeria	NGA	OOP	OOP	OOP	OOP	OOP	OOP	OOP	OOP	OOP	OOP	OOP	OOP	OOP	OOP	OOP	OOP	OOP	OOP
North Macedonia	MKD	SHI	SHI	SHI	SHI	SHI	SHI	SHI	SHI	SHI	SHI	SHI	SHI	SHI	SHI	SHI	SHI	SHI	SHI
Norway	NOR	GOV	GOV	GOV	GOV	GOV	GOV	GOV	GOV	GOV	GOV	GOV	GOV	GOV	GOV	GOV	GOV	GOV	GOV
Oman	OMN	GOV	GOV	GOV	GOV	GOV	GOV	GOV	GOV	GOV	GOV	GOV	GOV	GOV	GOV	GOV	GOV	GOV	GOV
Pakistan	PAK	OOP	OOP	OOP	OOP	OOP	OOP	OOP	OOP	OOP	OOP	OOP	OOP	OOP	OOP	OOP	OOP	OOP	OOP
Panama	PAN	GOV	OOP	OOP	OOP	OOP	OOP	OOP	OOP	OOP	OOP	OOP	OOP	OOP	OOP	OOP	OOP	OOP	OOP
Papua New Guinea	PNG	GOV	GOV	GOV	GOV	GOV	GOV	GOV	GOV	GOV	GOV	GOV	GOV	GOV	GOV	GOV	GOV	GOV	GOV
Paraguay	PRY	OOP	OOP	OOP	OOP	OOP	OOP	OOP	OOP	OOP	OOP	OOP	OOP	OOP	OOP	OOP	OOP	OOP	OOP
Peru	PER	OOP	OOP	OOP	OOP	OOP	OOP	OOP	OOP	OOP	OOP	OOP	OOP	OOP	OOP	OOP	OOP	OOP	OOP
Philippines	PHL	OOP	OOP	OOP	OOP	OOP	OOP	OOP	OOP	OOP	OOP	OOP	OOP	OOP	OOP	OOP	OOP	OOP	OOP
Poland	POL	SHI	SHI	SHI	SHI	SHI	SHI	SHI	SHI	SHI	SHI	SHI	SHI	SHI	SHI	SHI	SHI	SHI	SHI
Portugal	PRT	GOV	GOV	GOV	GOV	GOV	GOV	GOV	GOV	GOV	GOV	GOV	GOV	GOV	GOV	GOV	GOV	GOV	GOV
Qatar	QAT	GOV	GOV	GOV	GOV	GOV	GOV	GOV	GOV	GOV	GOV	GOV	GOV	GOV	GOV	GOV	GOV	GOV	GOV
Romania	ROU	SHI	SHI	SHI	SHI	SHI	SHI	SHI	SHI	SHI	SHI	SHI	SHI	SHI	SHI	SHI	SHI	SHI	SHI
Russian Federation	RUS	OOP	OOP	OOP	OOP	OOP	OOP	OOP	OOP	OOP	OOP	OOP	OOP	OOP	OOP	OOP	SHI	SHI	SHI
Rwanda	RWA	OOP	OOP	OOP	GOV	GOV	GOV	GOV	GOV	GOV	GOV	GOV	GOV	GOV	GOV	GOV	GOV	GOV	GOV
Senegal	SEN	OOP	OOP	OOP	OOP	OOP	OOP	OOP	OOP	OOP	OOP	OOP	OOP	OOP	OOP	OOP	OOP	OOP	OOP
Serbia	SRB	SHI	SHI	SHI	SHI	SHI	SHI	SHI	SHI	SHI	SHI	SHI	SHI	SHI	SHI	SHI	SHI	SHI	SHI
Sierra Leone	SLE	OOP	OOP	OOP	OOP	OOP	OOP	OOP	OOP	OOP	OOP	OOP	OOP	OOP	OOP	OOP	OOP	OOP	OOP
Singapore	SGP	OOP	OOP	OOP	OOP	OOP	OOP	OOP	OOP	OOP	OOP	OOP	OOP	OOP	OOP	OOP	OOP	OOP	OOP
Slovak Republic	SVK	SHI	SHI	SHI	SHI	SHI	SHI	SHI	SHI	SHI	SHI	SHI	SHI	SHI	SHI	SHI	SHI	SHI	SHI
Slovenia	SVN	SHI	SHI	SHI	SHI	SHI	SHI	SHI	SHI	SHI	SHI	SHI	SHI	SHI	SHI	SHI	SHI	SHI	SHI
South Africa	ZAF	GOV	GOV	GOV	GOV	GOV	GOV	GOV	GOV	GOV	GOV	GOV	GOV	GOV	GOV	GOV	GOV	GOV	GOV
Spain	ESP	GOV	GOV	GOV	GOV	GOV	GOV	GOV	GOV	GOV	GOV	GOV	GOV	GOV	GOV	GOV	GOV	GOV	GOV
Sri Lanka	LKA	GOV	GOV	GOV	GOV	GOV	GOV	GOV	OOP	OOP	OOP	OOP	OOP	OOP	OOP	OOP	OOP	OOP	OOP
Sudan	SDN	OOP	OOP	OOP	OOP	OOP	OOP	OOP	OOP	OOP	OOP	OOP	OOP	OOP	OOP	OOP	OOP	OOP	OOP
Sweden	SWE	GOV	GOV	GOV	GOV	GOV	GOV	GOV	GOV	GOV	GOV	GOV	GOV	GOV	GOV	GOV	GOV	GOV	GOV
Switzerland	CHE	SHI	SHI	SHI	SHI	SHI	SHI	SHI	SHI	SHI	SHI	SHI	SHI	SHI	SHI	SHI	SHI	SHI	SHI
Tajikistan	TJK	OOP	OOP	OOP	OOP	OOP	OOP	OOP	OOP	OOP	OOP	OOP	OOP	OOP	OOP	OOP	OOP	OOP	OOP
Tanzania	TZA	OOP	OOP	OOP	OOP	GOV	GOV	GOV	GOV	GOV	GOV	OOP	OOP	OOP	GOV	GOV	GOV	GOV	GOV
Thailand	THA	GOV	GOV	GOV	GOV	GOV	GOV	GOV	GOV	GOV	GOV	GOV	GOV	GOV	GOV	GOV	GOV	GOV	GOV
Togo	TGO	OOP	OOP	OOP	OOP	OOP	OOP	OOP	OOP	OOP	OOP	OOP	OOP	OOP	OOP	OOP	OOP	OOP	OOP
Trinidad and Tobago	TTO	OOP	OOP	OOP	OOP	OOP	OOP	OOP	OOP	OOP	OOP	OOP	OOP	OOP	GOV	GOV	GOV	GOV	GOV
Tunisia	TUN	OOP	OOP	OOP	OOP	OOP	OOP	OOP	OOP	OOP	OOP	OOP	OOP	OOP	OOP	OOP	OOP	OOP	OOP
Turkey	TUR	SHI	SHI	SHI	SHI	SHI	SHI	SHI	SHI	SHI	SHI	SHI	SHI	SHI	SHI	SHI	SHI	SHI	SHI
Turkmenistan	TKM	OOP	OOP	OOP	OOP	OOP	OOP	OOP	OOP	OOP	OOP	OOP	OOP	OOP	OOP	OOP	OOP	OOP	OOP
Uganda	UGA	OOP	OOP	OOP	OOP	OOP	OOP	OOP	OOP	OOP	OOP	OOP	OOP	OOP	OOP	OOP	OOP	OOP	OOP
Ukraine	UKR	OOP	OOP	OOP	GOV	GOV	GOV	GOV	GOV	GOV	GOV	GOV	GOV	GOV	OOP	OOP	OOP	OOP	OOP
United Arab Emirates	ARE	GOV	GOV	GOV	GOV	GOV	GOV	GOV	GOV	GOV	GOV	GOV	GOV	GOV	GOV	GOV	GOV	GOV	GOV
United Kingdom	GBR	GOV	GOV	GOV	GOV	GOV	GOV	GOV	GOV	GOV	GOV	GOV	GOV	GOV	GOV	GOV	GOV	GOV	GOV
United States	USA	OOP	OOP	OOP	OOP	OOP	OOP	SHI	SHI	SHI	SHI	SHI	SHI	SHI	SHI	SHI	SHI	SHI	SHI
Uruguay	URY	OOP	OOP	OOP	OOP	OOP	OOP	OOP	OOP	OOP	OOP	OOP	SHI	SHI	SHI	SHI	SHI	SHI	SHI
Uzbekistan	UZB	OOP	OOP	OOP	OOP	OOP	OOP	OOP	OOP	OOP	OOP	OOP	OOP	OOP	OOP	OOP	OOP	OOP	OOP
Venezuela, RB	VEN	OOP	OOP	OOP	OOP	OOP	OOP	GOV	GOV	OOP	OOP	OOP	OOP	OOP	OOP	OOP	OOP	OOP	OOP
Vietnam	VNM	OOP	OOP	OOP	OOP	OOP	OOP	OOP	OOP	OOP	OOP	OOP	OOP	OOP	OOP	OOP	OOP	OOP	OOP
Zambia	ZMB	GOV	GOV	GOV	GOV	GOV	OOP	OOP	OOP	OOP	GOV	GOV	GOV	GOV	GOV	GOV	GOV	GOV	GOV

*Note*: The table shows the predominant HFS for all countries, across years.

*Source*: Authors' elaboration.

In the below Table we present, for Year 2017 and all countries, SHI as % of THE, OOP as % of THE, government financing as % of THE, and voluntary health insurance as % of THE, the predominant HFS defined using clustering, and the predominant HFS defined as the HFS with the largest % of THE. Countries in which there is a difference between the two HFS definitions are noted in italic (9 cases, 6% of total country observations in 2017).

**Table:**

Country	Country code	Year	SHI as % THE	Government financing as % of THE	OOP as % of THE	VHI as % of THE	Predominance defined by clustering	Predominance as "largest as % of THE"
Angola	AGO	2017	0	47	34	6	Government financing predominant	Government financing predominant
United Arab Emirates	ARE	2017	0	72	19	8	Government financing predominant	Government financing predominant
Argentina	ARG	2017	43	30	15	9	SHI predominant	SHI predominant
Armenia	ARM	2017	0	14	84	1	OOP predominant	OOP predominant
Australia	AUS	2017	0	65	18	10	Government financing predominant	Government financing predominant
Austria	AUT	2017	44	30	19	5	SHI predominant	SHI predominant
Azerbaijan	AZE	2017	0	15	84	1	OOP predominant	OOP predominant
Burundi	BDI	2017	1	47	25	1	Government financing predominant	Government financing predominant
Belgium	BEL	2017	56	21	18	5	SHI predominant	SHI predominant
Benin	BEN	2017	2	39	45	6	OOP predominant	OOP predominant
Burkina Faso	BFA	2017	0	61	32	1	Government financing predominant	Government financing predominant
Bangladesh	BGD	2017	0	19	74	0	OOP predominant	OOP predominant
Bulgaria	BGR	2017	43	9	47	1	SHI predominant	OOP predominant
Bahrain	BHR	2017	0	58	31	11	Government financing predominant	Government financing predominant
Bosnia and Herzegovina	BIH	2017	68	2	29	0	SHI predominant	SHI predominant
Belarus	BLR	2017	0	70	28	1	Government financing predominant	Government financing predominant
Bolivia	BOL	2017	30	40	25	3	Government financing predominant	Government financing predominant
Brazil	BRA	2017	0	42	27	29	Government financing predominant	Government financing predominant
Bhutan	BTN	2017	0	79	13	0	Government financing predominant	Government financing predominant
Botswana	BWA	2017	0	78	3	9	Government financing predominant	Government financing predominant
Central African Republic	CAF	2017	0	19	31	0	OOP predominant	OOP predominant
Canada	CAN	2017	1	69	14	10	Government financing predominant	Government financing predominant
Switzerland	CHE	2017	42	22	29	7	SHI predominant	SHI predominant
Chile	CHL	2017	58	2	34	6	SHI predominant	SHI predominant
China	CHN	2017	38	18	36	5	SHI predominant	SHI predominant
Cote d'Ivoire	CIV	2017	2	35	39	8	OOP predominant	OOP predominant
Cameroon	CMR	2017	0	18	71	6	OOP predominant	OOP predominant
Congo, Dem. Rep.	COD	2017	1	30	40	3	OOP predominant	OOP predominant
Congo, Rep.	COG	2017	0	36	48	1	OOP predominant	OOP predominant
Colombia	COL	2017	68	6	16	10	SHI predominant	SHI predominant
Comoros	COM	2017	3	16	75	1	OOP predominant	OOP predominant
Costa Rica	CRI	2017	72	3	21	3	SHI predominant	SHI predominant
Cuba	CUB	2017	0	89	10	0	Government financing predominant	Government financing predominant
Cyprus	CYP	2017	0	42	45	12	OOP predominant	OOP predominant
Czech Republic	CZE	2017	69	13	15	0	SHI predominant	SHI predominant
Germany	DEU	2017	78	6	13	1	SHI predominant	SHI predominant
Djibouti	DJI	2017	11	43	27	0	Government financing predominant	Government financing predominant
Denmark	DNK	2017	0	84	14	2	Government financing predominant	Government financing predominant
Dominican Republic	DOM	2017	25	21	45	8	OOP predominant	OOP predominant
Algeria	DZA	2017	26	40	33	1	OOP predominant	Government financing predominant
Ecuador	ECU	2017	24	29	39	6	OOP predominant	OOP predominant
Egypt, Arab Rep.	EGY	2017	4	29	60	1	OOP predominant	OOP predominant
Eritrea	ERI	2017	0	38	59	0	OOP predominant	OOP predominant
Spain	ESP	2017	4	66	24	5	Government financing predominant	Government financing predominant
Estonia	EST	2017	64	10	24	0	SHI predominant	SHI predominant
Ethiopia	ETH	2017	0	45	34	1	Government financing predominant	Government financing predominant
Finland	FIN	2017	14	62	20	2	Government financing predominant	Government financing predominant
Fiji	FJI	2017	0	67	16	13	Government financing predominant	Government financing predominant
France	FRA	2017	78	5	9	7	SHI predominant	SHI predominant
Gabon	GAB	2017	24	39	25	9	Government financing predominant	Government financing predominant
United Kingdom	GBR	2017	0	79	16	3	Government financing predominant	Government financing predominant
Georgia	GEO	2017	0	37	55	6	OOP predominant	OOP predominant
Ghana	GHA	2017	10	30	40	2	OOP predominant	OOP predominant
Guinea	GIN	2017	2	29	57	1	OOP predominant	OOP predominant
Gambia, the	GMB	2017	0	41	22	4	Government financing predominant	Government financing predominant
Guinea‐Bissau	GNB	2017	1	14	72	0	OOP predominant	OOP predominant
Equatorial Guinea	GNQ	2017	1	19	77	0	OOP predominant	OOP predominant
Guatemala	GTM	2017	17	18	54	4	OOP predominant	OOP predominant
Guyana	GUY	2017	2	61	32	2	Government financing predominant	Government financing predominant
Honduras	HND	2017	12	33	49	5	OOP predominant	OOP predominant
Croatia	HRV	2017	76	6	11	4	SHI predominant	SHI predominant
Haiti	HTI	2017	2	12	40	5	OOP predominant	OOP predominant
Hungary	HUN	2017	61	8	27	2	SHI predominant	SHI predominant
Indonesia	IDN	2017	23	26	35	4	OOP predominant	OOP predominant
India	IND	2017	5	23	62	5	OOP predominant	OOP predominant
Ireland	IRL	2017	0	73	12	13	Government financing predominant	Government financing predominant
Iran, Islamic Rep.	IRN	2017	32	14	42	7	OOP predominant	OOP predominant
Israel	ISR	2017	48	16	22	11	SHI predominant	SHI predominant
Italy	ITA	2017	0	74	23	2	Government financing predominant	Government financing predominant
Jamaica	JAM	2017	6	61	17	16	Government financing predominant	Government financing predominant
Jordan	JOR	2017	16	34	30	15	OOP predominant	Government financing predominant
Japan	JPN	2017	76	9	13	2	SHI predominant	SHI predominant
Kazakhstan	KAZ	2017	0	62	33	1	Government financing predominant	Government financing predominant
Kenya	KEN	2017	8	42	24	10	Government financing predominant	Government financing predominant
Kyrgyz Republic	KGZ	2017	7	35	56	0	OOP predominant	OOP predominant
Cambodia	KHM	2017	0	23	60	1	OOP predominant	OOP predominant
Korea, Rep.	KOR	2017	49	10	34	7	SHI predominant	SHI predominant
Kuwait	KWT	2017	0	87	13	0	Government financing predominant	Government financing predominant
Lao PDR	LAO	2017	2	36	46	0	OOP predominant	OOP predominant
Lebanon	LBN	2017	24	25	33	16	OOP predominant	OOP predominant
Liberia	LBR	2017	0	32	46	7	OOP predominant	OOP predominant
Sri Lanka	LKA	2017	0	44	50	2	OOP predominant	OOP predominant
Lesotho	LSO	2017	0	68	17	0	Government financing predominant	Government financing predominant
Lithuania	LTU	2017	58	9	32	1	SHI predominant	SHI predominant
Latvia	LVA	2017	0	57	42	1	Government financing predominant	Government financing predominant
Morocco	MAR	2017	20	25	54	1	OOP predominant	OOP predominant
Moldova	MDA	2017	50	2	44	0	SHI predominant	SHI predominant
Madagascar	MDG	2017	0	54	25	3	Government financing predominant	Government financing predominant
Mexico	MEX	2017	28	24	41	6	OOP predominant	OOP predominant
North Macedonia	MKD	2017	63	5	32	0	SHI predominant	SHI predominant
Mali	MLI	2017	10	34	35	1	OOP predominant	OOP predominant
Myanmar	MMR	2017	1	17	76	0	OOP predominant	OOP predominant
Mongolia	MNG	2017	23	41	32	0	OOP predominant	Government financing predominant
Mozambique	MOZ	2017	2	52	7	2	Government financing predominant	Government financing predominant
Mauritania	MRT	2017	10	34	50	2	OOP predominant	OOP predominant
Mauritius	MUS	2017	0	43	49	6	OOP predominant	OOP predominant
Malawi	MWI	2017	0	50	11	3	Government financing predominant	Government financing predominant
Malaysia	MYS	2017	1	50	38	10	Government financing predominant	Government financing predominant
Namibia	NAM	2017	0	48	8	38	Government financing predominant	Government financing predominant
Niger	NER	2017	1	44	48	1	OOP predominant	OOP predominant
Nigeria	NGA	2017	1	14	77	0	OOP predominant	OOP predominant
Nicaragua	NIC	2017	24	40	33	1	OOP predominant	Government financing predominant
Netherlands	NLD	2017	75	6	11	6	SHI predominant	SHI predominant
Norway	NOR	2017	0	85	14	0	Government financing predominant	Government financing predominant
Nepal	NPL	2017	0	25	58	1	OOP predominant	OOP predominant
New Zealand	NZL	2017	9	69	14	5	Government financing predominant	Government financing predominant
Oman	OMN	2017	0	88	7	3	Government financing predominant	Government financing predominant
Pakistan	PAK	2017	1	29	60	1	OOP predominant	OOP predominant
Panama	PAN	2017	28	33	33	6	OOP predominant	OOP predominant
Peru	PER	2017	30	33	28	7	OOP predominant	Government financing predominant
Philippines	PHL	2017	12	23	53	11	OOP predominant	OOP predominant
Papua New Guinea	PNG	2017	0	76	9	0	Government financing predominant	Government financing predominant
Poland	POL	2017	59	10	23	6	SHI predominant	SHI predominant
Portugal	PRT	2017	1	65	28	4	Government financing predominant	Government financing predominant
Paraguay	PRY	2017	17	28	44	10	OOP predominant	OOP predominant
Qatar	QAT	2017	0	81	9	9	Government financing predominant	Government financing predominant
Romania	ROU	2017	63	15	20	1	SHI predominant	SHI predominant
Russian Federation	RUS	2017	36	21	40	2	SHI predominant	OOP predominant
Rwanda	RWA	2017	17	52	6	2	Government financing predominant	Government financing predominant
Sudan	SDN	2017	11	8	72	1	OOP predominant	OOP predominant
Senegal	SEN	2017	4	34	52	8	OOP predominant	OOP predominant
Singapore	SGP	2017	8	40	32	3	OOP predominant	Government financing predominant
Sierra Leone	SLE	2017	0	27	50	0	OOP predominant	OOP predominant
El Salvador	SLV	2017	29	35	29	6	OOP predominant	Government financing predominant
Serbia	SRB	2017	54	3	42	0	SHI predominant	SHI predominant
Slovak Republic	SVK	2017	78	2	19	0	SHI predominant	SHI predominant
Slovenia	SVN	2017	69	3	12	14	SHI predominant	SHI predominant
Sweden	SWE	2017	0	84	15	1	Government financing predominant	Government financing predominant
Eswatini	SWZ	2017	0	49	10	11	Government financing predominant	Government financing predominant
Chad	TCD	2017	0	21	58	4	OOP predominant	OOP predominant
Togo	TGO	2017	3	25	58	7	OOP predominant	OOP predominant
Thailand	THA	2017	11	68	11	7	Government financing predominant	Government financing predominant
Tajikistan	TJK	2017	0	33	63	0	OOP predominant	OOP predominant
Turkmenistan	TKM	2017	0	22	73	5	OOP predominant	OOP predominant
Trinidad and Tobago	TTO	2017	0	53	40	7	Government financing predominant	Government financing predominant
Tunisia	TUN	2017	31	27	39	3	OOP predominant	OOP predominant
Turkey	TUR	2017	56	22	17	2	SHI predominant	SHI predominant
Tanzania	TZA	2017	8	62	24	1	Government financing predominant	Government financing predominant
Uganda	UGA	2017	0	20	39	2	OOP predominant	OOP predominant
Ukraine	UKR	2017	0	45	52	1	OOP predominant	OOP predominant
Uruguay	URY	2017	50	17	18	11	SHI predominant	SHI predominant
United States	USA	2017	58	26	11	0	SHI predominant	SHI predominant
Uzbekistan	UZB	2017	0	45	53	0	OOP predominant	OOP predominant
Venezuela, RB	VEN	2017	6	10	63	21	OOP predominant	OOP predominant
Vietnam	VNM	2017	23	27	45	1	OOP predominant	OOP predominant
South Africa	ZAF	2017	0	43	8	36	Government financing predominant	Government financing predominant
Zambia	ZMB	2017	0	56	12	1	Government financing predominant	Government financing predominant

## APPENDIX 4. Full baseline results, and heterogeneity within HFS groups

**Table:**

	(1)	(2)	(3)	(4)	(5)	(6)
	Log THE per capita	Imm, index	LE	Log U5M	Log MM	CHE10
Variables	FE	FE	FE	FE	FE	FE
Predominant government financing	0.043	3.804	1.341**	−0.083**	−0.040	−3.256***
	(0.041)	(2.921)	(0.579)	(0.036)	(0.040)	(0.931)
Predominant SHI	0.117***	−1.486	−0.128	0.051	0.034	6.467***
	(0.035)	(1.606)	(0.395)	(0.037)	(0.067)	(1.129)
Log GDP per capita (PPP)	0.698***	3.591	1.038	−0.315***	−0.515***	1.230
	(0.093)	(3.776)	(0.643)	(0.097)	(0.178)	(2.073)
Corruption control	0.099**	3.783*	−0.087	−0.058	0.029	2.662**
	(0.041)	(2.225)	(0.577)	(0.043)	(0.079)	(1.140)
Government effectiveness	0.030	−1.636	0.125	−0.088**	−0.041	1.968
	(0.044)	(1.874)	(0.350)	(0.038)	(0.068)	(2.521)
% of population above 65	0.000	−0.389	−0.263*	0.022*	0.049	−0.020
	(0.019)	(0.954)	(0.156)	(0.013)	(0.035)	(0.453)
% population below 14	−0.017*	0.645**	0.335***	−0.029***	−0.035**	0.224
	(0.009)	(0.321)	(0.074)	(0.008)	(0.016)	(0.235)
Urbanization (%)	−0.009	0.364**	0.073	0.003	0.005	0.175
	(0.006)	(0.164)	(0.055)	(0.006)	(0.013)	(0.176)
Access to drinking water (% population)	−0.003	0.474	0.150***	−0.007	0.002	0.074
	(0.005)	(0.298)	(0.042)	(0.004)	(0.012)	(0.080)
Enrollment to primary school (gross, % population	−0.002	0.035	0.019	0.002	−0.001	−0.080*
	(0.002)	(0.075)	(0.017)	(0.001)	(0.002)	(0.040)
Gini index	0.005	0.259*	−0.009	0.004	0.006	0.018
	(0.004)	(0.152)	(0.035)	(0.003)	(0.004)	(0.099)
Country FE	YES	YES	YES	YES	YES	YES
Year FE	YES	YES	YES	YES	YES	YES
Adjusted R2	0.869	0.177	0.752	0.879	0.646	0.224
Observations	950	970	970	970	970	407
Number of countries	124	124	124	124	124	111

Notes: FE estimates are the result of Equation (1). Robust SEs, clustered at country‐level, in parentheses. Details on HFS switches are detailed in Appendix 3. All models control for all variables listed as “control” in Table 2. *p*‐values for two‐sided *t*‐tests are reported as: ****p* < 0.01, ***p* < 0.05, **p* < 0.1.

*Source*: Author elaboration.

The results below are for the following equation: $Y_{it}=\alpha +\rho_{1}{\mathrm{SHI}\%\mathrm{THE}}_{it}+\rho_{2}{\mathrm{GOV}\%\mathrm{THE}}_{it}+\rho_{3}{\mathrm{OOP}\%\mathrm{THE}}_{it}+\gamma X_{it}+T_{t}+C_{i}+\varepsilon_{it}$, for the subsample of country‐year observations that belong to the HFS‐predominant group noted in the column of each model, for column 1–18. For column 19–36, only SHI as percentage of THE is used for the SHI‐predominant HFS group, OOP expenditures as percentage of THE is used for the OOP‐predominant HFS group, and government financing as % of THE is used for the government financing predominant HFS group.

**Table:**

	(1)	(2)	(3)	(4)	(5)	(6)	(7)	(8)	(9)	(10)	(11)	(12)	(13)	(14)	(15)	(16)	(17)	(18)
	Ln THE	Imm Idx	LE	ln U5M	ln MM	CAT 10%	Ln THE	Imm Idx	LE	ln U5M	ln MM	CAT 10%	Ln THE	Imm Idx	LE	ln U5M	ln MM	CAT 10%
	FE	FE	FE	FE	FE	FE	FE	FE	FE	FE	FE	FE	FE	FE	FE	FE	FE	FE
HFS group → variables ↓	GOV group	GOV group	GOV group	GOV group	GOV group	GOV group	OOP group	OOP group	OOP group	OOP group	OOP group	OOP group	SHI group	SHI group	SHI group	SHI group	SHI group	SHI group
Government financing %	−0.002	0.000	0.049	−0.000	0.002	0.001*	−0.001	0.001	−0.018	0.003	−0.004	−0.003*	0.003	0.001	−0.034**	0.001	0.005	−0.001
	(0.006)	(0.001)	(0.051)	(0.003)	(0.005)	(0.001)	(0.004)	(0.002)	(0.025)	(0.003)	(0.003)	(0.002)	(0.003)	(0.001)	(0.013)	(0.003)	(0.005)	(0.002)
SHI financing %	−0.005	0.004	0.151	0.009	0.019*	−0.000	0.012***	−0.001	−0.007	0.005	−0.004	−0.001	−0.002	−0.001	−0.014	0.000	0.004	0.000
	(0.013)	(0.002)	(0.093)	(0.007)	(0.011)	(0.002)	(0.004)	(0.002)	(0.026)	(0.003)	(0.004)	(0.001)	(0.002)	(0.001)	(0.011)	(0.002)	(0.005)	(0.000)
OOP financing %	−0.003	0.001	0.030	−0.001	−0.006	0.002***	−0.000	0.001	0.010	0.005*	−0.003	−0.002	0.002	−0.000	0.021	−0.001	0.011	0.001
	(0.006)	(0.002)	(0.051)	(0.003)	(0.008)	(0.001)	(0.004)	(0.002)	(0.022)	(0.003)	(0.002)	(0.001)	(0.002)	(0.002)	(0.015)	(0.003)	(0.007)	(0.001)
Observations	265	271	271	271	271	116	398	407	407	407	407	193	287	292	292	292	292	98
R‐squared	0.875	0.145	0.811	0.940	0.842	0.528	0.875	0.359	0.816	0.922	0.733	0.404	0.959	0.281	0.925	0.873	0.745	0.400
Number of ID	45	46	46	46	46	39	69	69	69	69	69	60	31	31	31	31	31	23
Country FE	YES	YES	YES	YES	YES	YES	YES	YES	YES	YES	YES	YES	YES	YES	YES	YES	YES	YES
Year FE	YES	YES	YES	YES	YES	YES	YES	YES	YES	YES	YES	YES	YES	YES	YES	YES	YES	YES
Adjusted R^2	0.861	0.0417	0.788	0.933	0.823	0.376	0.865	0.310	0.802	0.916	0.713	0.302	0.954	0.202	0.917	0.859	0.716	0.168

	(19)	(20)	(21)	(22)	(23	(24)	(25)	(26)	(27)	(28)	(29)	(30)	(31)	(32)	(33)	(34)	(35)	(36)
	Ln THE	Imm Idx	LE	ln U5M	ln MM	CAT 10%	Ln THE	Imm Idx	LE	ln U5M	ln MM	CAT 10%	Ln THE	Imm Idx	LE	ln U5M	ln MM	CAT 10%
	FE	FE	FE	FE	FE	FE	FE	FE	FE	FE	FE	FE	FE	FE	FE	FE	FE	FE
Variables	GOV group	GOV group	GOV group	GOV group	GOV group	GOV group	OOP group	OOP group	OOP group	OOP group	OOP group	OOP group	CHI group	CHI group	CHI group	CHI group	CHI group	CHI group
Government financing %	0.000	−0.001	0.020	0.000	0.004	0.000												
	(0.003)	(0.001)	(0.035)	(0.002)	(0.004)	(0.000)												
SHI financing %							−0.003	−0.001	−0.008	−0.000	−0.001	0.000						
							(0.002)	(0.001)	(0.009)	(0.002)	(0.003)	(0.000)						
OOP financing %													−0.003	0.001	0.022	0.002	0.000	−0.000
													(0.003)	(0.001)	(0.016)	(0.001)	(0.002)	(0.001)
Observations	265	271	271	271	271	116	287	292	292	292	292	98	398	407	407	407	407	193
R‐squared	0.875	0.142	0.808	0.939	0.835	0.418	0.958	0.280	0.918	0.873	0.733	0.379	0.858	0.353	0.815	0.920	0.730	0.337
Number of ID	45	46	46	46	46	39	31	31	31	31	31	23	69	69	69	69	69	60
Country FE	YES	YES	YES	YES	YES	YES	YES	YES	YES	YES	YES	YES	YES	YES	YES	YES	YES	YES
Year FE	YES	YES	YES	YES	YES	YES	YES	YES	YES	YES	YES	YES	YES	YES	YES	YES	YES	YES
Adjusted R^2	0.861	0.0463	0.787	0.932	0.817	0.248	0.954	0.207	0.910	0.860	0.705	0.163	0.848	0.307	0.802	0.914	0.710	0.233

*Notes*: this table present the results of the fixed effects (FE) Equation (1), restricting the sample to the sub‐group of “countries within the government‐, SHI‐ or OOP‐predominant group”. All the controls variables and FE (time and country FE) used in the main models are also used here. The independent variable of interest is the percentage of THE channelled via government schemes, SHI schemes and OOP schemes. In the first table, all three percentages have been used. In the second table, only the percentage of THE channelled via OOP schemes has been used for the OOP‐predominant group, the percentage of THE channelled via SHI schemes for the SHI‐predominant group, and the percentage of THE channelled via government schemes for the government‐predominant group.

*Source*: Author elaboration. Datasets discussed in Section 4.

## APPENDIX 5. FE estimates of augmented model with interaction terms (Equation 4), Section 5.3. Results in italic when observations are not enough.

**Table:**

	(1)	(2)	(3)	(4)	(5)	(6)	(7)	(8)	(9)	(10)	(11)	(12)
Interaction terms ↓ // Dependent variables →	Imm. Index	LE	ln U5M	ln MM	CAT 10%	GGHE % GGE	Imm index	LE	ln U5M	ln MM	CAT 10%	GGHE % GGE
Government‐predominant & informal sector size	−0.198*	−0.009	0.000	0.002	1.585***	0.034						
	(0.098)	(0.021)	(0.002)	(0.002)	(0.005)	(0.047)						
SHI‐predominant & informal sector size	−0.221**	−0.001	0.006***	−0.002		−0.021						
	(0.088)	(0.014)	(0.002)	(0.002)		(0.048)						
Government‐predominant & logged GDP per capita (PPP)							−0.470	0.372	−0.090*	−0.174	1.341	−0.693
							(2.491)	(0.559)	(0.046)	(0.119)	(0.820)	(0.478)
SHI‐predominant & logged GDP per capita (PPP)							5.847**	−0.260	−0.006	−0.007	−2.469	0.220
							(2.367)	(0.454)	(0.058)	(0.081)	(2.045)	(0.604)
Observations	184	184	184	184	50	184	970	970	970	970	407	970
Number of ID	34	34	34	34	26	34	124	124	124	124	111	124
Country FE	YES	YES	YES	YES	YES	YES	YES	YES	YES	YES	YES	YES
Year FE	YES	YES	YES	YES	YES	YES	YES	YES	YES	YES	YES	YES
Adjusted *R* ^2^	0.216	0.851	0.952	0.844	1	0.474	0.199	0.754	0.880	0.648	0.189	0.172

	(13)	(14)	(15)	(16)	(17)	(18)	(19)	(20)	(21)	(22)	(23)	(24)
Interaction terms ↓//Dependent variables →	Imm. Index	LE	ln U5M	ln MM	CAT 10%	GGHE % GGE	Imm index	LE	ln U5M	ln MM	CAT 10%	GGHE % GGE
Government‐predominant & government effectiveness	1.662	1.391*	−0.113*	−0.167	−0.132	0.412						
	(3.198)	(0.714)	(0.063)	(0.127)	(2.943)	(0.752)						
SHI‐predominant & government effectiveness	−1.362	−0.229	−0.073	0.006	−2.920	0.309						
	(3.867)	(0.540)	(0.052)	(0.112)	(4.440)	(0.816)						
Government‐predominant & control of corruption							−2.015	1.659	−0.038	−0.174	−1.612	0.785
							(2.575)	(1.119)	(0.062)	(0.116)	(2.124)	(0.666)
SHI‐predominant & control of corruption							−0.215	−0.277	−0.038	0.038	−4.538	1.00***
							(2.330)	(0.319)	(0.033)	(0.069)	(3.359)	(0.328)
Observations	970	970	970	970	407	970	970	970	970	970	407	970
Number of ID	124	124	124	124	111	124	124	124	124	124	111	124
Country FE	YES	YES	YES	YES	YES	YES	YES	YES	YES	YES	YES	YES
Year FE	YES	YES	YES	YES	YES	YES	YES	YES	YES	YES	YES	YES
Adjusted *R* ^2^	0.189	0.753	0.879	0.645	0.178	0.166	0.188	0.753	0.880	0.646	0.195	0.171

	(25)	(26)	(27)	(28)	(29)	(30)	(31)	(32)	(33)	(34)	(35)	(36)
Interaction terms ↓//Dependent variables →	Imm Idx	LE	ln U5M	ln MM	CAT 10%	GGHE % GGE	Imm Idx	LE	ln U5M	ln MM	CAT 10%	GGHE % GGE
Government‐predominant & GGE as % of GDP	−0.036	0.012	0.001	−0.003	−0.055	−0.085**						
	(0.163)	(0.030)	(0.002)	(0.004)	(0.090)	(0.038)						
SHI‐predominant & GGE as % of GDP	0.084	0.013	0.005	−0.005	−0.172	−0.055						
	(0.230)	(0.041)	(0.004)	(0.005)	(0.202)	(0.047)						
Government‐predominant & labour‐tax as % of health revenues							−0.015	0.035	−0.000	−0.002	−0.218	0.057**
							(0.21)	(0.036)	(0.003)	(0.003)	(0.240)	(0.024)
SHI‐predominant & labour‐tax as % of health revenues							0.174	−0.021	−0.005	−0.002	−0.019	−0.114***
							(0.12)	(0.027)	(0.003)	(0.005)	(0.106)	(0.042)
Observations	970	970	970	970	407	970	970	970	970	970	407	970
Number of ID	124	124	124	124	111	124	124	124	124	124	111	124
Country FE	YES	YES	YES	YES	YES	YES	YES	YES	YES	YES	YES	YES
Year FE	YES	YES	YES	YES	YES	YES	YES	YES	YES	YES	YES	YES
Adjusted *R* ^2^	0.191	0.756	0.881	0.649	0.179	0.244	0.192	0.755	0.881	0.646	0.184	0.213

*Note*: Datasets discussed in Section 4. This table present the results of the fixed effects (FE) with interaction terms, Equation (2). The interaction terms coefficient and *p*‐values are shown in the table. Results are in italics when observations are below 100.

*Source*: Author elaboration.

## APPENDIX 6. Robustness checks

**TABLE A1:** Robustness checks

	Intermediate outcomes		HEALTH system OUTCOMES			
	(1)	(2)	(3)	(4)	(5)	(6)
	Log THE per capita	Imm. coverage	LE	Log U5M	Log MM	CAT 10%
	FE	FE	FE	FE	FE	FE
PANEL A: BASELINE ESTIMATES						
Predominant government	0.043	3.804	1.341**	−0.083**	−0.040	−3.256***
	(0.041)	(2.921)	(0.579)	(0.036)	(0.040)	(0.931)
Predominant SHI	0.117***	−1.486	−0.128	0.051	0.034	6.467***
	(0.035)	(1.606)	(0.395)	(0.037)	(0.067)	(1.129)
PANEL B: LMICs ONLY						
Predominant government	0.040	3.416	1.366**	−0.073*	−0.042	−3.305***
	(0.043)	(3.133)	(0.608)	(0.037)	(0.038)	(0.923)
Predominant SHI	0.127***	−1.195	−0.220	0.093**	0.019	6.273***
	(0.038)	(2.337)	(0.497)	(0.039)	(0.051)	(1.297)
PANEL C: REMOVED OUTLIERS						
Predominant government	0.018	2.894	1.062***	−0.066*	−0.010	−2.764***
	(0.041)	(1.780)	(0.323)	(0.035)	(0.032)	(0.954)
Predominant SHI	0.119***	−1.766	−0.079	0.049	0.032	6.312***
	(0.036)	(1.552)	(0.398)	(0.037)	(0.067)	(1.094)
PANEL D: LAGGED HFS						
Predominant government	−0.0104	3.210	0.785*	−0.075**	−0.0148	−3.016***
	(−0.27)	(−0.99)	(−1.94)	(−2.07)	(−0.36)	(−3.35)
Predominant SHI	0.105***	−1.183	−0.170	0.062	0.0446	5.917***
	(−2.84)	(−0.63)	(−0.41)	(−1.24)	(−0.65)	(−6.04)
PANEL E: GENERAL GOVERNMENT EXPENDITURE (%GDP) ADDED AS CONTROL						
Predominant government	0.038	3.790	1.359**	−0.082**	−0.042	−3.312***
	(0.043)	(2.917)	(0.570)	(0.038)	(0.042)	(0.944)
Predominant SHI	0.099***	−1.537	−0.066	0.056	0.025	6.512***
	(0.037)	(1.596)	(0.374)	(0.036)	(0.066)	(1.123)
PANEL F: USE PERCENTAGES						
% government	−0.000	0.022	−0.004	−0.001	0.001	−0.130***
	(0.002)	(0.084)	(0.018)	(0.001)	(0.003)	(0.045)
% SHI	0.006**	−0.033	−0.014	0.002	0.002	0.036
	(0.003)	(0.091)	(0.020)	(0.002)	(0.004)	(0.043)
PANEL G: DIFFERENTIAL (THE, U5M) AND RANDOM TREND (LE, CAT 10%) MODELS						
Predominant government	0.051		0.129	−0.077**		−1.102
	(0.036)		(0.134)	(0.032)		(1.092)
Predominant SHI	0.142***		−0.114	0.040		0.121
	(0.033)		(0.112)	(0.039)		(0.585)
PANEL H: NOT LOGGED MORTALITY OUTCOMES						
Predominant government				−9.496**	−52.003	
				(4.066)	(33.701)	
Predominant SHI				2.335	8.218	
				(1.425)	(7.773)	
PANEL I: REMOVE GINI INDEX CONTROL VARIABLE TO MAXIMIZE NUMBER OF OBSERVATIONS						
Predominant government	0.014	0.984	0.979***	−0.065**	−0.048	−1.814
	(0.037)	(1.556)	(0.350)	(0.029)	(0.037)	(1.226)
Predominant SHI	0.095**	−2.759*	−0.540*	−0.016	−0.025	4.583***
	(0.047)	(1.665)	(0.307)	(0.040)	(0.056)	(0.996)
PANEL J: REMOVE CONTROLS TO HAVE ALL 147 COUTNRIES IN SAMPLE						
Predominant government	0.010	1.861	0.840**	−0.041	−0.032	−1.566
	(0.034)	(1.938)	(0.337)	(0.025)	(0.032)	(1.237)
Predominant SHI	0.086*	−4.079**	−1.205***	0.001	0.049	3.523***
	(0.046)	(1.718)	(0.325)	(0.044)	(0.076)	(1.210)
PANEL K: ADD CONFOUNDERS ADJUSTMENT (ZELDOW AND HATFIELD, 2021)						
Predominant government	0.038	5.416*	1.132***	−0.061*	−0.042	−2.141**
	(0.036)	(2.844)	(0.305)	(0.034)	(0.031)	(1.024)
Predominant SHI	0.049	0.622	−0.299	0.071	0.089	4.167*
	(0.034)	(2.275)	(0.254)	(0.048)	(0.069)	(2.283)
PANEL L: ADD DEVELOPMENT ASSISTANCE FOR HEALTH (% THE) AS A CONTROL						
Predominant government	0.050	3.691	1.254**	−0.082**	−0.038	−3.298***
	(0.041)	(2.936)	(0.510)	(0.036)	(0.039)	(0.944)
Predominant SHI	0.126***	−1.657	−0.260	0.053	0.036	6.427***
	(0.037)	(1.670)	(0.377)	(0.037)	(0.067)	(1.132)
PANEL M: USE LOG GDP DECILES AS CONTROL VARIABLE, INSTEAD OF LOG GDP						
Predominant government	0.024	3.595	1.248**	−0.077**	−0.032	−3.047***
	(0.048)	(3.035)	(0.557)	(0.036)	(0.046)	(0.970)
Predominant SHI	0.155***	−1.247	−0.048	0.035	0.007	6.517***
	(0.041)	(1.650)	(0.369)	(0.038)	(0.063)	(1.071)

*Note*: Datasets discussed in Section 4. Models detail explained in the robustness checks section. Baseline model controls variable included in all models unless specified. In italic, in panel G, coefficients that are statistically different from the baseline model at the 10% level based on tests in Equations (4) and (9). Robust standard errors in parenthesis.

****p* < 0.01, ***p* < 0.05, **p* < 0.1.

*Source*: Author elaboration.

**TABLE A2:** Estimates using different definitions of “predominant HFS”

	Intermediate OUTCOMES		HEALTH system outcomes			
	(1)	(2)	(3)	(4)	(5)	(6)
	Log THE per capita	Imm. coverage	LE	Log U5M	LogMM	CAT 10%
	FE	FE	FE	FE	FE	FE
PANEL A: BASELINE ESTIMATES (HFS DEFINED BY CLUSTERING)						
Predominant government	0.043	3.804	1.341**	−0.083**	−0.040	−3.256***
	(0.041)	(2.921)	(0.579)	(0.036)	(0.040)	(0.931)
Predominant SHI	0.117***	−1.486	−0.128	0.051	0.034	6.467***
	(0.035)	(1.606)	(0.395)	(0.037)	(0.067)	(1.129)
PANEL B: HFS DEFINED BY HIGHEST VALUE						
Government financing (% of THE) highest value	0.009	1.332	0.474	−0.075***	−0.060	−1.700*
	(0.038)	(1.253)	(0.296)	(0.022)	(0.040)	(0.872)
SHI Financing (% of THE) highest value	0.041	0.210	−0.467*	0.050	0.114***	3.782***
	(0.042)	(1.306)	(0.250)	(0.057)	(0.041)	(1.133)
PANEL C: OOP PREDOMINANT ONLY IF OOP‐%‐THE ABOVE 50%						
Predominant government	0.101	14.911*	1.020***	−0.053	−0.094	−1.916
	(0.065)	(8.554)	(0.359)	(0.037)	(0.076)	(1.801)
Predominant SHI	0.373***	−3.737**	0.934**	−0.048	−0.033	4.420**
	(0.056)	(1.805)	(0.418)	(0.039)	(0.065)	(2.092)
PANEL D: OOP PREDOMINANT ONLY IF OOP‐%‐THE ABOVE 45%						
Predominant government	−0.015	8.722**	0.519	−0.013	−0.032	−2.840***
	(0.049)	(3.799)	(0.341)	(0.025)	(0.043)	(1.082)
Predominant SHI	0.200***	−3.056**	0.424	0.043	−0.038	4.495***
	(0.031)	(1.193)	(0.452)	(0.036)	(0.055)	(1.431)
PANEL E: OOP PREDOMINANT ONLY IF OOP‐%‐THE ABOVE 40%						
Predominant government	−0.036	8.990**	0.818*	−0.023	−0.051	−2.547***
	(0.045)	(3.522)	(0.489)	(0.024)	(0.034)	(0.938)
Predominant SHI	0.189***	−3.508***	0.290	0.053	−0.022	5.295***
	(0.025)	(1.071)	(0.467)	(0.052)	(0.059)	(1.249)
PANEL F: OOP PREDOMINANT ONLY IF OOP‐%‐THE ABOVE 35%						
Predominant government	0.032	3.813	0.757*	−0.054	0.011	−2.136**
	(0.036)	(3.615)	(0.430)	(0.039)	(0.034)	(0.883)
Predominant SHI	0.163***	−3.411***	0.244	0.049	0.001	4.996***
	(0.028)	(1.297)	(0.438)	(0.066)	(0.046)	(1.354)
PANEL G: OOP PREDOMINANT ONLY IF OOP‐%‐THE ABOVE 30%						
Predominant government	0.047	4.360*	0.985***	−0.081***	−0.025	−2.691***
	(0.034)	(2.424)	(0.284)	(0.029)	(0.028)	(0.866)
Predominant SHI	0.139***	−2.883**	0.186	0.065	−0.024	6.548***
	(0.033)	(1.369)	(0.382)	(0.046)	(0.048)	(1.164)
PANEL H: USE ALL HF ARRANGEMENTS AS INPUT VARIABLES FOR CLUSTERING						
Predominant government	0.030	4.922	1.103**	−0.058**	−0.058*	−2.188**
	(0.033)	(3.400)	(0.448)	(0.027)	(0.034)	(0.856)
Predominant SHI	0.091***	−0.541	−0.227	0.060**	0.039	4.590***
	(0.027)	(1.487)	(0.350)	(0.029)	(0.056)	(1.333)
PANEL I: USE ALL HF ARRANGEMENTS and LN(THE PER CAPITA) AS INPUT VARIABLES FOR CLUSTERING						
Predominant government	0.030	5.289	1.067**	−0.055**	−0.053	−2.188**
	(0.033)	(3.527)	(0.466)	(0.028)	(0.036)	(0.856)
Predominant SHI	0.091***	−0.763	−0.235	0.057**	0.033	4.590***
	(0.027)	(1.408)	(0.355)	(0.026)	(0.053)	(1.333)
PANEL J: SET THE NUMBER OF CLUSTERS TO FOUR						
Predominant government	−0.005	1.571	0.545	−0.041	−0.101	−3.032*
	(0.067)	(4.615)	(0.810)	(0.043)	(0.103)	(1.604)
Predominant OOP, but less predominant	0.072	−1.390	−0.324	0.028	−0.023	−1.633
	(0.054)	(1.138)	(0.403)	(0.028)	(0.067)	(1.371)
Predominant SHI	0.146**	−1.474	−0.831	0.097**	0.065	3.205**
	(0.072)	(2.268)	(0.679)	(0.042)	(0.098)	(1.534)

*Note*: Datasets discussed in Section 4. Models detail explained in the robustness checks section. Baseline model controls variable included in all models unless specified. Robust standard errors in parenthesis.

****p* < 0.01, ***p* < 0.05, **p* < 0.1.

*Source*: Author elaboration.

**TABLE A3:** Robustness to different income sub‐groups

	Intermediate OUTCOMES		HEALTH system outcomes			
	(1)	(2)	(3)	(4)	(5)	(6)
	Log THE per capita	Imm. coverage	LE	Log U5M	Log MM	CAT 10%
	FE	FE	FE	FE	FE	FE
PANEL A: BASELINE ESTIMATES						
Predominant government	0.043	3.804	1.341**	−0.083**	−0.040	−3.256***
	(0.041)	(2.921)	(0.579)	(0.036)	(0.040)	(0.931)
Predominant SHI	0.117***	−1.486	−0.128	0.051	0.034	6.467***
	(0.035)	(1.606)	(0.395)	(0.037)	(0.067)	(1.129)
PANEL B: SUB‐GROUP OF HIGH‐ AND MIDDLE‐ INCOME COUNTRIES						
Predominant government	0.012	5.657*	0.903**	−0.062	−0.021	−2.657***
	(0.034)	(3.250)	(0.385)	(0.039)	(0.039)	(0.854)
Predominant SHI	0.090**	0.111	−0.246	0.061**	0.074	5.268***
	(0.041)	(1.921)	(0.432)	(0.029)	(0.066)	(1.000)
PANEL C: SUB‐GROUP OF LOW‐ AND MIDDLE‐INCOME COUNTRIES						
Predominant government	0.040	3.416	1.366**	−0.073*	−0.042	−3.305***
	(0.043)	(3.133)	(0.608)	(0.037)	(0.038)	(0.923)
Predominant SHI	0.127***	−1.195	−0.220	0.093**	0.019	6.273***
	(0.038)	(2.337)	(0.497)	(0.039)	(0.051)	(1.297)
PANEL D: SUB‐GROUP OF MIDDLE‐INCOME COUNTRIES						
Predominant government	−0.002	5.587	0.891**	−0.043	−0.018	−3.071***
	(0.035)	(3.554)	(0.404)	(0.039)	(0.042)	(0.891)
Predominant SHI	0.119***	−0.341	−0.062	0.087**	0.018	5.665***
	(0.041)	(2.524)	(0.539)	(0.037)	(0.057)	(1.398)
PANEL E: INCLUDE ALL COUNTRIES						
Predominant government	−0.012	−3.902	−0.787	0.047	0.039	2.120***
	(0.040)	(3.538)	(0.480)	(0.032)	(0.044)	(0.740)
Predominant SHI	0.091*	−4.297	−1.204*	0.126**	0.124	7.728***
	(0.054)	(3.222)	(0.708)	(0.049)	(0.087)	(1.213)
PANEL F: INCLUDE ALL COUNTRIES, USE HIGHEST VALUE FOR HFS DEFINITION						
Government financing (% of THE) highest value	0.009	1.356	0.521*	−0.083***	−0.065	−1.700*
	(0.038)	(1.255)	(0.303)	(0.023)	(0.041)	(0.871)
SHI Financing (% of THE) highest value	0.043	0.026	−0.473*	0.048	0.113***	3.782***
	(0.041)	(1.290)	(0.252)	(0.057)	(0.041)	(1.133)

*Note*: Equation (1) is estimated using different income sub‐groups, including all countries available in the dataset, and finally including all countries and using a different definition for HFS (highest value), as detailed in the robustness checks section and in Appendix 2. *Source*: Authors' elaboration. Datasets discussed in section 4.

**TABLE A4:** Additional outcomes

				Government‐predominant HFS		SHI‐predominant HFS						
	#	Dep. Variable	Model	$\rho_{2}$	SE	ρ1	SE	Obs.	Coun‐tries	Coun. FE	Year FE	Adj. *R* ^2^
HEALTH SYSTEM OUTCOMES	(1)	Ln U5M, DHS	FE	−0.080**	0.036	1.120***	0.119	292	59	YES	YES	0.045
	(2)	Ln IM, DHS	FE	−0.113**	0.042	0.969***	0.114	291	59	YES	YES	0.040
	(3)	AM, female	FE	−19.3**	(9.417)	−5.898	(8.310)	968	124	YES	YES	0.607
	(4)	AM, male	FE	−20.1**	(8.485)	−0.909	(10.533)	968	124	YES	YES	0.506
	(5)	MMR, national	FE	−182.28	(119.50)	−10.011	(11.235)	262	124	YES	YES	0.521
	(6)	CAT 25%	FE	−0.877***	(0.264)	0.899**	(0.438)	407	111	YES	YES	0.184
	(7)	Inc. Imp 3.10	FE	−0.380**	(0.163)	−0.735***	(0.231)	407	111	YES	YES	0.140
	(8)	Inc. Imp 1.90	FE	−0.220	(0.183)	−0.084	(0.182)	407	111	YES	YES	0.315
INTERMEDIATE OUTCOMES	(9)	SBA %	FE	1.208	(1.818)	−2.501***	(0.770)	629	94	YES	YES	0.471
	(10)	GGHE % GGE	FE	0.895**	(0.408)	1.047**	(0.472)	970	124	YES	YES	0.167
	(11)	THE % GDP	FE	0.332	(0.298)	0.847**	(0.349)	950	124	YES	YES	0.290

*Note*: Equation (1) is estimated using additional outcomes, as detailed in the Results section. For DHS outcomes, fewer controls were used because otherwise the number of observations would drop below 100. The controls used were: GDP per capita, urbanization, control of corruption, government effectiveness, % population above 65 and % population below 14 years old, in addition to fixed effects. For DHS outcomes, under‐5 mortality and infant mortality is provided as “average for the 5 and 10‐year period before the survey”: when it was 5‐year, the observation year was recorded as “survey year minus three”, when it was 10 years, the year considered was “survey year minus five”. In other words, we attach the “past 5 and 10 years average reading” to the mid‐year of that same 5 or 10 year period. Robust standard errors reported, clustered at country‐level.

P < 0.1*, *p* < 0.05**, *p* < 0.01***.

*Source*: Author elaboration.

*Notes*: these are event studies plots, for five outcomes out of six outcomes used for our main results. For CAT 10%, data limitations do not allow an event study. In the case of government financing, there are 30 countries switching between OOP and government financing predomaning HFSs. However, only 17 countries switch only once during the 2000–2018 study period. These 17 countries are included in the event study, while the remaining 13 countries who switch back‐and‐forth between OOP and government financing are excluded, as the interpretation of their coefficients is not possible.

The equation used for the above plots is: $Y_{it}=\alpha +\sum_{j=0}^{5}\rho_{j}{GOV}_{it-j}+\rho_{2}{GOV}_{it+2}+\gamma X_{it}+T_{t}+C_{i}+\varepsilon_{it}$

Adding further leads resulted in omitted variables, therefore we limited leads to only 2 years before the switch from OOP to government financing.

The baseline omitted case is the first lead, where *k* = 1.

*Notes*: these are event studies plots, for five outcomes out of six outcomes used for our main results. For CAT 10%, data limitations do not allow an event study. In the case of government financing, there are eight countries switching between OOP and SHI predomaning HFSs. Seven countries switch only once during the 2000–2018 study period. These seven countries are included in the event study, while the remaining 1 countries who switch back‐and‐forth between OOP and SHI are excluded, as the interpretation of their coefficients is not possible.

The equation used for the above plots is: $Y_{it}=\alpha +\sum_{j=0}^{5}\rho_{j}{SHI}_{it-j}+\sum_{k=2}^{3}\rho_{k}{SHI}_{it+k}+\gamma X_{it}+T_{t}+C_{i}+\varepsilon_{it}$

The baseline omitted case is the first lead, where *k* = 1.
